# TNF-alpha-induced microglia activation requires miR-342: impact on NF-kB signaling and neurotoxicity

**DOI:** 10.1038/s41419-020-2626-6

**Published:** 2020-06-02

**Authors:** João Paulo Brás, Joana Bravo, Jaime Freitas, Mário Adolfo Barbosa, Susana Gomes Santos, Teresa Summavielle, Maria Inês Almeida

**Affiliations:** 1 i3S - Instituto de Investigação e Inovação em Saúde, University of Porto, Rua Alfredo Allen 208, 4200-135 Porto, Portugal; 2 INEB - Instituto de Engenharia Biomédica, University of Porto, Rua Alfredo Allen 208, 4200-135 Porto, Portugal; 3 ICBAS - Instituto de Ciências Biomédicas Abel Salazar, University of Porto, Rua de Jorge Viterbo Ferreira 228, 4050-313 Porto, Portugal; 4 IBMC - Instituto de Biologia Molecular e Celular, University of Porto, Rua Alfredo Allen 208, 4200-135 Porto, Portugal; 5 ESS - Escola Superior de Saúde, Instituto Politécnico do Porto, Rua Dr. António Bernardino de Almeida 400, 4200-072 Porto, Portugal

**Keywords:** Neuroimmunology, miRNAs

## Abstract

Growing evidences suggest that sustained neuroinflammation, caused by microglia overactivation, is implicated in the development and aggravation of several neurological and psychiatric disorders. In some pathological conditions, microglia produce increased levels of cytotoxic and inflammatory mediators, such as tumor necrosis factor alpha (TNF-α), which can reactivate microglia in a positive feedback mechanism. However, specific molecular mediators that can be effectively targeted to control TNF-α-mediated microglia overactivation, are yet to be uncovered. In this context, we aim to identify novel TNF-α-mediated micro(mi)RNAs and to dissect their roles in microglia activation, as well as to explore their impact on the cellular communication with neurons. A miRNA microarray, followed by RT-qPCR validation, was performed on TNF-α-stimulated primary rat microglia. Gain- and loss-of-function in vitro assays and proteomic analysis were used to dissect the role of miR-342 in microglia activation. Co-cultures of microglia with hippocampal neurons, using a microfluidic system, were performed to understand the impact on neurotoxicity. Stimulation of primary rat microglia with TNF-α led to an upregulation of *Nos2*, *Tnf*, and *Il1b* mRNAs. In addition, ph-NF-kB p65 levels were also increased. miRNA microarray analysis followed by RT-qPCR validation revealed that TNF-α stimulation induced the upregulation of miR-342. Interestingly, miR-342 overexpression in N9 microglia was sufficient to activate the NF-kB pathway by inhibiting BAG-1, leading to increased secretion of TNF-α and IL-1β. Conversely, miR-342 inhibition led to a strong decrease in the levels of these cytokines after TNF-α activation. In fact, both TNF-α-stimulated and miR-342-overexpressing microglia drastically affected neuron viability. Remarkably, increased levels of nitrites were detected in the supernatants of these co-cultures. Globally, our findings show that miR-342 is a crucial mediator of TNF-α-mediated microglia activation and a potential target to tackle microglia-driven neuroinflammation.

## Introduction

In recent years, the immune system has been associated with pathological events occurring in several neurodegenerative and psychiatric disorders^1^. Accordingly, growing evidence shows that some of the patients suffering from these disorders have chronic microglia activation and overproduction of pro-inflammatory cytokines, such as tumor necrosis factor alpha (TNF-α), which sustains several neuroinflammatory processes^2^.

Microglia are the largest population of immune cells in the central nervous system (CNS), corresponding to 5–15% of all adult brain^3^. Under physiological conditions, these cells are located within the brain parenchyma, where they are in direct contact with neural progenitors, neurons, and other glial cells (namely astrocytes and oligodendrocytes)^4^. When an injury or infection takes place, microglia are recruited to the site where they engulf invading pathogens and extracellular debris by phagocytosis, supporting the normal function and integrity of the brain^5^. In response to an inflammatory stimulus, microglia secrete a number of molecules such as proteinases, nitric oxide, reactive oxygen intermediates and pro-inflammatory cytokines, including interleukin-1 beta (IL-1β), interleukin-6 (IL-6), and TNF-α^6,7^.

TNF-α is a major Th1-class pro-inflammatory cytokine, produced by a variety of immune cells. In the CNS, activated microglia are one of the main sources of TNF-α^8,9^. TNF-α can bind to TNFR1(p55) and/or TNFR2(p75), activating downstream signaling pathways that mediate a wide variety of biological responses, including apoptosis, cell differentiation, proliferation, survival, and inflammation^10^. At basal levels, TNF-α has an important role in brain development, particularly by influencing hippocampal development and function^11^. However, in certain pathological conditions, increased levels of this cytokine overactivate microglia, which then causes neuronal damage, such as demyelination and/or neuronal degeneration. Overactivated microglia release cytotoxic molecules, including TNF-α, which is produced by a positive feedback mechanism of autocrine activation^12,13^. Although the basic mechanisms by which TNF-α activates microglia have been previously reported, the specific molecular mediators that can be effectively targeted to control TNF-α-mediated microglia overactivation and neuroinflammation, are still to be uncovered.

In the last decade, microRNAs (miRNAs) have been extensively studied owing to their regulatory role in many pathological events^14,15^. miRNAs are short, single-stranded, non-protein coding RNAs, which regulate gene expression at the post-transcriptional level by inducing mRNA translation inhibition or degradation^16^. Importantly, miRNAs are highly conserved in mammalians^17^ and are crucial players in key cellular processes, such as inflammation, cell death, and differentiation. Remarkably, dysfunction in one single miRNA can concomitantly impair several biological functions by acting on distinct mRNA targets, as previously described by us^16–20^. In addition, miRNAs can be encapsulated into extracellular vesicles, and mediate cell-to-cell communication^19,21^. All these features make miRNAs attractive candidates for use as biomarkers and/or as therapeutic targets.

Herein, we investigated the role of miRNAs in TNF-α-driven microglia activation, in order to identify potential therapeutic targets for microglia chronic activation and neuroinflammation. Specifically, we aimed to unravel how TNF-α impacted miRNAs expression, to identify which of these miRNAs are essential for microglia activation, ultimately determining their impact on the cross-talk between microglia and neurons.

## Materials and methods

### Animal ethical disclosure

All procedures to obtain primary cell cultures were conducted in accordance with European regulations (European Union Directive 2010/63/EU) and were approved by the i3S Animal Ethics Committee and the Portuguese regulatory entity—Direcção Geral de Alimentação e Veterinária (DGAV, ref 11769/2014-05-15 to TS). Animal facilities and the people directly involved in animal experiments (J.P.B. and J.B.) were also certified. All efforts were made to ensure minimal animal suffering, and to follow the principles of the 3Rs.

### Cell culture

#### Mixed glial cells

In brief, newborn Wistar Han rats (P1–P2) were decapitated and brains separated from the skull. Cerebellum and meninges were carefully removed for efficient brain dissection. Dissected tissue was treated with DNase and trypsin (Sigma, USA) before being dissociated and plated. Rat brain-derived mixed glial cells were cultured in T75 poly-d-lysine (Sigma) coated flasks for 21 days. Culture was maintained in DMEM (Dulbecco’s modified Eagle’s medium; Gibco, USA) supplemented with 10% fetal bovine serum (FBS; Biowest, France).

#### Primary microglia

Primary microglia were isolated from rat mixed glial cultures using the shaking method, as previously described^22^. Microglia were obtained after 14 and 21 days of culture by shaking the flasks at 37 °C for 2 h at 150 rpm. Isolated microglia were re-seeded (3 × 10^4^/cm^2^) in DMEM/F12 (Corning, USA) supplemented with 10% FBS, allowed to adhere for 48 h, and then stimulated for 6 h with 100 ng/mL lipopolysaccharide (LPS, Sigma) or 20 ng/mL TNF-α (Peprotech, UK).

#### Primary hippocampal neurons

E17 C57BL/6 mice hippocampal neurons were cultured as previously described^23^. In brief, after dissection, hippocampi were treated with trypsin (1.5 mg/mL, 15 min, 37 °C, Sigma) in Hank’s balanced salt solution (HBSS; Gibco), washed with HBSS containing 10% FBS, to stop trypsin activity, and washed in HBSS to remove serum and avoid glia growth. Finally, the tissue was transferred to serum-free Neurobasal medium (Gibco), supplemented with B27 (1:50, Gibco), glutamine (0.5 mm, Sigma), gentamycin (0.12 mg/mL Gibco), and glutamate (25 μm, Sigma), and dissociated mechanically. Neurons were then plated (1 × 10^5^ cells/chamber) in poly-d-lysine-coated coverslips (20 μg/cm^2^) previously attached to Axon Investigation Systems (AXIS, AX150, Millipore) and maintained in the supplemented neurobasal medium. Cells were kept at 37 °C in a humidified incubator with 5% CO_2_/95% air, for 14 days, and half of the media was replaced at day 7.

#### N9 microglial cells

Murine N9 microglia cells, kindly donated by professor João Relvas (i3S, Porto), were cultured in RPMI 1640 (Corning, USA) supplemented with 10% FBS in T75 flasks. All cell lines were tested and negative for mycoplasma contamination. Prior to transfections, cells were trypsinized, re-seeded (1.5 × 10^4^/cm^2^) and allowed to adhere for 24 h. 70% confluent N9 microglia cells were transfected with mirVana miRNA mimic/mirVana miRNA inhibitor for mmu-miR-342–3p, or respective controls (mirVana miRNA mimic/inhibitor negative controls; Invitrogen, USA), using Lipofectamine 2000 (Invitrogen), as recommended by the manufacturer. After transfection, when applicable, N9 microglia cells were stimulated with 20 ng/mL of TNF-α for 6 h. Conditioned media was collected for cytokine quantification and cells harvested for protein extraction or co-culture with neurons. Transfections with siRNA were also performed with Lipofectamine 2000. siRNA specific for murine BAG-1 (5′-CCGUUGUCAGCACUUGGAAUGCAAA-3’) and SiRNA negative control (12,935–300) were purchased from Invitrogen. Overexpression of BAG-1 in N9 microglia was achieved by transiently transfecting the cells with a BAG-1 mammalian expression vector (pCMV6-BAG-1, Origene). In brief, 24 hours before transfection, cells were seeded into six-well cell culture plates at a density of 1.2 × 10^5^ cells per well in regular growth medium. Transfections were performed with Lipofectamine 2000 according to the manufacturer’s instruction (Invitrogen). In all, 48 hours after transfection, cells were harvested for the detection of ph-NF-kB p65 expression levels by western blot. The control vector pCMV6 was kindly donated by Mariana Santos (UnIGENe, i3S, Portugal).

#### Primary hippocampal neurons and N9 microglial cells co-culture

Co-cultures were performed in Axon Investigation System (Millipore). At day 13 of neuronal culture, transfected, TNF-α or non-stimulated N9 microglia cells (0.2 × 10^5^ cells) were added to each Axon Investigation System, in direct contact with axons for 24 h. Culture media was then collected for nitrites quantification and cells were fixed prior to immunostaining.

### Flow cytometry

Primary microglia culture purity was measured by flow cytometry using the following antibodies: mouse anti-rat CD11b/c-PE/Cy7 (BD Biosciences, USA) and mouse anti-rat CD45-FITC (ImmunoTools, Germany). Unlabeled microglia, mouse isotypes IgG2a-PE/Cy7 (BD Biosciences) and IgG2a-FITC (Immunoools) were used as negative controls. Fluorescence was measured in a FACS Canto II flow cytometer (BD Biosciences) with BD FACS Diva software. Results were analyzed using FlowJo Software.

### RNA extraction

Total RNA was extracted using TRIzol reagent (Invitrogen) according to the manufacturer’s instructions. RNA concentration and purity were evaluated in a NanoDrop 1000 (Thermo Scientific). Ratios of 260/280 and 260/230 nm ranged between 1.9 and 2.1. RNA integrity was evaluated by agarose gel electrophoresis or by Experion automated electrophoresis system (Bio-Rad, USA).

### Reverse transcription and real-time quantitative polymerase chain reaction

For gene expression analysis, RNA was treated with TURBO DNA-free Kit (Invitrogen) and cDNA was synthesized using Random Hexamers (Invitrogen), dNTPs (Bioline) and SuperScript III Reverse Transcriptase (Invitrogen). qPCR was carried out in CFX96 Touch Real-Time PCR Detection System (Bio-Rad, USA) using cDNA, primers and iQ SYBR Green Supermix (Bio-Rad). Oligonucleotides used for qPCR experiments are shown in Supplementary Table 1.

miR-146b-5p, miR-342–3p, miR-124–3p, and let-7i-5p expression was evaluated using TaqMan miRNA assays (Applied Biosystems). In brief, cDNA was synthesized using 30 ng of RNA as a template, gene-specific stem-loop Reverse Transcription primer, and the TaqMan miRNA reverse transcription kit (Applied Biosystems). qPCR was carried out in CFX96 Touch Real-Time PCR Detection System (Bio-Rad) using cDNA, TaqMan probe and SsoAdvanced Universal Probes Supermix (Bio-Rad). Small nuclear RNA U6 was used as reference gene. All runs were performed in duplicate. Relative expression levels from eight independent experiments were calculated using the quantification cycle (C_q_) method, according to MIQE guidelines^24^.

### miRNA microarray assay

miRNA expression profile of TNF-α-activated primary rat microglia was performed through ArrayStar, using a μParaflo mouse miRNA microarray, Array 19.0 (LC Sciences, USA). By adding a poly (A) tail to the 3’ end with poly (A) polymerase, total RNA (2 μg/sample) was extended, and then an oligonucleotide tag was ligated to the poly (A) tail for fluorescent dye staining subsequently. Prepared RNA was hybridized to a μParaflo microfluidic chip. Detection probes were synthesized in situ, based on miRBase v22.0 database (www.mirbase.org)^25^. Following hybridization, the Cy3 dye was bound to the oligo tag for staining through the microfluidic chip. Fluorescence images were acquired with a laser scanner and digitized with Array-Pro image analysis software. Data relative to the fluorescent intensity of a given hybridization target (signal) is presented in arbitrary units. Only miRNAs with average signals ≥20 and −0.2 ≤ log_2_ fold change to CTR ≥ 0.2 were considered for further evaluation (Supplementary Table 3).

### Western blot

N9 microglia cells were harvested and washed twice with cold PBS before lysis in the presence of protease and phosphatase inhibitors. Cell lysates were centrifuged (14,000 rpm, 10 min, 4 °C) and total protein quantified using DC protein assay kit (Bio-Rad, USA). Protein samples were resolved by SDS-PAGE in reducing conditions and transferred to nitrocellulose membranes, which were blocked in a solution of 5% BSA in TBS-Tween 0.1%. Membranes were then probed using the following primary antibodies: anti-ph-NF-kB p65, anti-BAG-1, anti-α-Tubulin, and anti-glyceraldehyde 3-phosphate dehydrogenase (GAPDH). Appropriate secondary antibodies conjugated to horseradish peroxidase were used for signal detection. Antibodies manufacturers and respective dilutions are indicated in Supplementary Table 2. Protein expression levels were quantified using ImageLab. α-Tubulin and GAPDH were used as normalizers.

### Analysis of nuclear NF-kB translocation by imaging flow cytometry

Murine N9 microglial cells were stimulated with 20 ng/mL of TNF-α for 10, 20, or 30 minutes or transfected with the miRNA mimic mmu-miR-342–3p and the miRNA mimic negative control (SCR), as described. Before harvesting, cells were washed twice with ice-cold PBS and fixed in 4% PFA. NF-kB staining with rabbit anti-mouse Phospho-NF-κB p65 (Ser536) (93H1) antibody (Cell Signaling Technologies) was performed in PBS with 0.1% Triton X-100 and 2% FBS for 20 min on ice, followed by a 20 min incubation with anti-rabbit Alexa Fluor 488-labeled secondary antibody (Thermo Fisher Scientific) in PBS with 2% FBS. Nuclei were stained with 20 mm DRAQ5 (Biostatus) for 10 min before acquisition. Cells were acquired using an ImageStreamX Imaging flow cytometer with the INSPIRE software and equipped with an Extended Depth of Field filter (Amnis, EMD Millipore), at the Bioimaging Center for Biomaterials and Regenerative Therapies (b.IMAGE – i3S, Porto). Data analysis was performed with IDEAS software (Amnis, EMD Millipore). Fluorescence compensation was performed with single-stained samples. For each sample 30,000 cells were acquired, and >10,000 single, in focus, double positive for NF-kB and DRAQ5 cells were analyzed for NF-kB translocalization. The IDEAS software nuclear translocation wizard was used to determine the similarity coefficient between the NF-kB and DRAQ5 (nuclei) staining’s. As described by AM Silva et al.^26^, nuclear translocation was considered for NF-kB/DRAQ5 similarity coefficients above 1.

### Protein identification by Nanoscale Liquid Chromatography coupled to tandem Mass Spectrometry (nano LC-MS/MS)

Protein identification and quantitation was performed by nano LC-MS/MS. This equipment is composed by an Ultimate 3000 liquid chromatography system coupled to a Q-Exactive Hybrid Quadrupole-Orbitrap mass spectrometer (Thermo Scientific, Germany). Samples were loaded onto a trapping cartridge for 3 min and further separated in a nano-C18 column at 300 nL/min. Data acquisition was controlled by Xcalibur and Tune software (Thermo Scientific). The mass spectrometer was operated in data-dependent (dd) positive acquisition mode alternating between a full scan (m/z 380–1580) and subsequent HCD MS/MS of the 10 most intense peaks from full scan. Raw data were processed using Proteome Discoverer 2.2.0.388 software (Thermo Scientific). Protein identification was performed with Sequest HT search engine against the *Mus musculus* entries from the UniProt database.

### Enzyme-linked immunosorbent assay (ELISA)

Supernatants of N9 microglial cells were collected and processed (1500 rpm, 10 min, 4 °C). TNF-α, Il-1β, IL-6, MIP-2, IL-12, IL-10, and IL-4 levels were evaluated by ELISA, according to the manufacturer’s instructions (ABTS ELISA Development Kit, PeproTech). Cytokine levels were measured in a plate reader at 405 nm, with wavelength correction at 650 nm. Cytokine concentrations (pg/mL) were determined using a standard calibration curve.

### Immunofluorescence

Primary neurons and N9 microglial cells were washed and fixed with 4% paraformaldehyde (PFA) in PBS. Cells were permeabilized with 0.25% Triton in PBS prior to blocking and overnight incubation at 4 °C with primary antibodies: mouse anti-β3-Tubulin (Biolegend) and rabbit anti-Iba1 (Wako) for neurons and microglia, respectively. Secondary antibodies anti-mouse Alexa 488 (Cell Signaling Technologies) and anti-rabbit Alexa 594 (Invitrogen) were incubated for 1 h at RT. Nuclear staining was performed by incubating cells with Hoechst (Sigma) for 5 min at RT. Coverslips were mounted in microscope slides with Fluoroshield (Sigma) and images randomly acquired in a Zeiss Axio Imager Z1 Apotome. Neuronal apoptosis was addressed by evaluating nuclei shape of ten images per condition^27^.

### Nitrites quantification (Griess assay)

Supernatants from neuron-N9 microglia co-cultures were mixed with an equal volume of Griess reagent in a 96-well plate. Sodium nitrite (1000 nm, Sigma) was serial diluted to generate the standard curve. Absorbance was read at 550 nm and nitrites concentration calculated using a standard curve.

### Statistical analysis

Statistical analysis was performed using GraphPad Prism version 7 (GaphPad Software, Inc.). Gaussian distribution was tested by the Shapiro-Wilk normality test. For non-normal distribution data, tests were used to evaluate significant differences between samples, namely Wilcoxon matched-pairs signed rank test (between two groups) and Friedman test, followed by uncorrected Dunn’s multiple comparison test (more than two groups). When the data passed normality tests, one-way analysis of variance (more than two groups), followed by Sidak’s multiple comparison test was used. The statistical test used is identified in each figure legend. Experiments were performed at least three times independently. All samples were included in the analysis. Statistical significance was considered for *p* < 0.05 (*p < 0.05, **p < 0.01, ***p < 0.001, n.s.: non-significant).

## Results

### TNF-α induces microglia activation through NF-kB

**Fig. 1:**
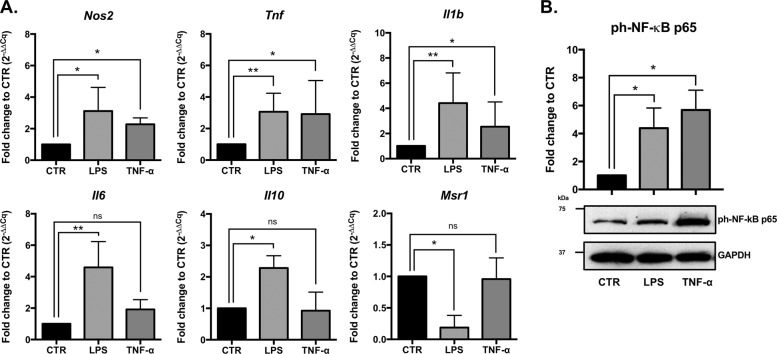
TNF-α-induced overexpression of pro-inflammatory genes, through activation of NF-kB pathway in microglia. Rat microglia were obtained from mixed glial cultures. Isolated microglia were re-seeded in six-well plates, allowed to adhere for 48 h and then stimulated for 6 h with LPS (100 ng/mL) or TNF-α (20 ng/mL). **a** Gene expression profile of activated microglia evaluated by RT-qPCR. Results were normalized with *Gapdh* and are expressed in fold change to CTR (mean ± SD, *n* = 5). **b** NF-kB p65 phosphorylation levels evaluation by western blot after microglia activation with LPS or TNF-α (mean ± SD, *n* = 5). GAPDH was used as normalizer. Statistical significance: **p < 0.01, *p < 0.05, ns–non significant; Friedman test followed by Dunn’s multiple comparisons test.

To better understand the mechanism by which TNF-α activates microglia, primary rat mixed glial cells (microglia, oligodendrocytes, and astrocytes) were isolated from P1–P2 rats. Microglia was obtained using the well-described shaking method, which allowed the setup of microglial in vitro experiments with a cell purity >99% (Supplementary Figs. 1 and 2). To assess the impact of TNF-α on the expression of inflammation-associated genes, microglia was stimulated with 20 ng/mL of recombinant TNF-α for 6 h, determined as the peak of NF-kB p65 phosphorylation (Supplementary Figs 3). LPS (100 ng/mL) was used as a positive control of microglia activation. We observed that TNF-α significantly induced the upregulation of *Nos2* (mean fold change to CTR (FC) = 2.22, *p* = 0.027), *Tnf* (FC = 2.92, *p* = 0.014) and *Il1b* (FC = 2.54, *p* = 0.026) pro-inflammatory mRNAs compared with non-stimulated microglia (Fig. 1a). Interestingly, TNF-α had no impact in the expression of *Il-6*, *Il10* and *Msr1* mRNAs, whereas LPS induced the upregulation of *Il6* (FC = 4.69, *p* = 0.0016) and *Il10* (FC = 2.28, *p* = 0.027) and downregulation of *Msr1* (FC = 0.19, *p* = 0.027; Fig. 1a). Importantly, we also observed that TNF-α-induced microglia activation resulted in increased levels of ph-NF-kB p65 (*p* = 0.011; Fig. 1b), suggesting that the overexpression of the inflammatory mRNAs may be achieved through the activation of the NF-kB pathway.

### miR-342 is overexpressed in TNF-α-activated microglia

**Fig. 2:**
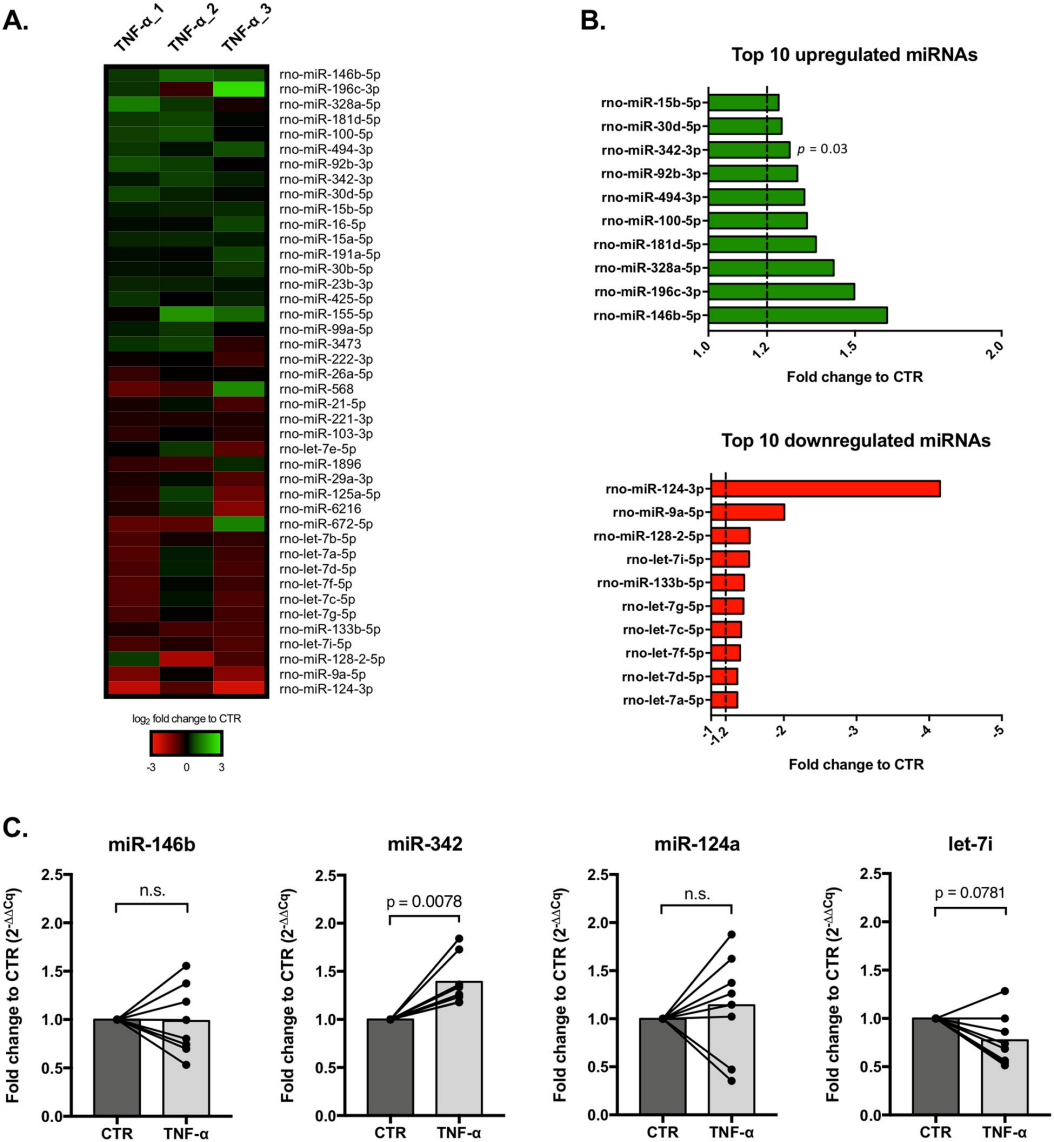
miR-342 is overexpressed in TNF-α-stimulated microglia. **a** Heat map of miRNA microarray expression results in TNF-α activated microglia (*n* = 3). Only miRNAs with −0.2 ≤ log_2_ FC to CTR ≥ 0.2 and a detection signal ≥20 are represented. **b** Ten most upregulated (green) or downregulated (red) miRNAs in TNF-α stimulated microglia (mean FC to CTR, *n* = 3). **c** Fold change to control based on the relative expression of the selected miRNAs, evaluated by RT-qPCR (mean, *n* = 8). U6 SnRNA was used as reference. Cq: quantification cycle. Statistical significance: *p* < 0.05; Wilcoxon matched-pairs test.

In order to explore the role of miRNAs in TNF-α-driven microglia activation, a miRNA microarray was performed in three independent experiments (Fig. 2a). Only miRNAs averaging a detection signal >20 were considered. Nineteen miRNAs were upregulated (log_2_ FC to CTR > 0.2), whereas 23 were downregulated (log_2_ FC to CTR < −0.2) in microglia stimulated with TNF-α compared with control cells (Supplementary Table 3). miR-146b-5p was the most expressed miRNA (FC to CTR = 1.61), however, only miR-342–3p was significantly upregulated (FC to CTR = 1.28, *p* = 0.03; Fig. 2b and Supplementary Table 3). miR-124–3p, the most downregulated miRNA (FC to CTR = –4.15; Fig. 2b and Supplementary Table 3), and several members of the let-7 family members were all downregulated on TNF-α-activated microglia (Fig. 2b), although not statistically different to control. To validate these results, miRNA expression levels were evaluated by RT-qPCR. In fact, microarray results were confirmed, as only miR-342–3p was significantly upregulated after TNF-α stimulation (FC to CTR = 1.39; *p* = 0.0078; *n* = 8). Neither miR-146b-5p, miR-124–3p, nor let-7i-5p (a member of let-7 family) were significantly altered (Fig. 2c). These results suggest that miR-342 may have a role in TNF-α-mediated microglia activation.

### miR-342 has a role in TNF-α-mediated microglia activation

**Fig. 3:**
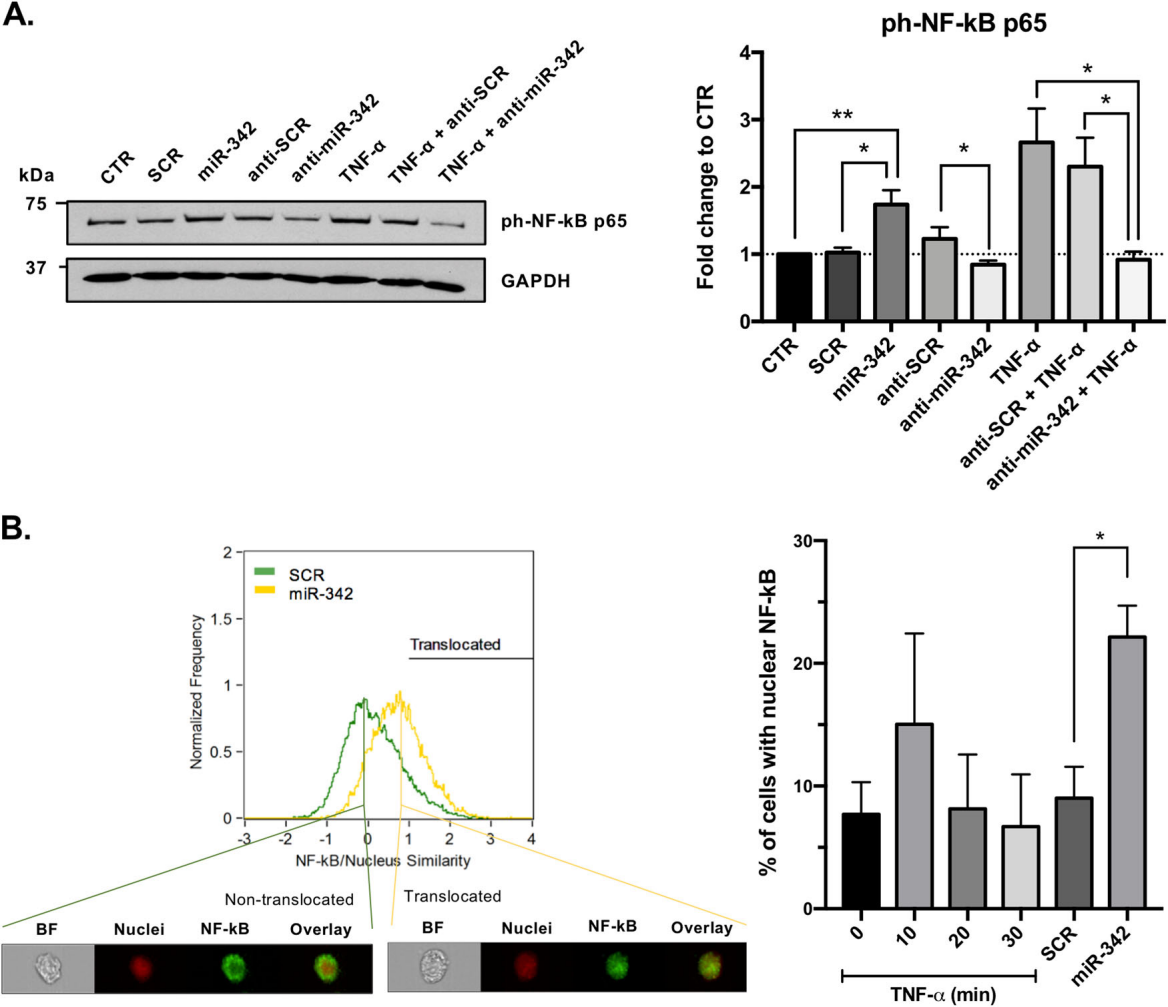
miR-342 regulates TNF-α-mediated microglia activation through NF-kB. **a** ph-NF-kB p65 expression evaluation by western blot after TNF-α stimulation and/or mirVana miRNA mimic/inhibitor mmu-miR-342–3p or mirVana miRNA mimic/inhibitor negative control (SCR) transfection. Results were normalized with GAPDH and compared with non-stimulated N9 microglia (mean ± SD, *n* = 3–6). **b** Representative plot of the similarity coefficient between NF-kB and nuclei staining’s in cells transfected with mirVana miRNA mimic negative control (SCR, green) or miRNA mimic mmu-miR-342–3p (miR-342, yellow). The black line (Translocated) corresponds to gated cells with nuclear translocated NF-kB (similarity coefficient >1). Representative images of cells with a similarity coefficient <1 (non-translocated) and with a similarity coefficient >1 (translocated) are shown below. *BF* brightfield. On the right, graph shows the quantification of the percentage of cells with nuclear translocated NF-κB (translocated gate, similarity coefficient >1) after exposure to TNF-α for the indicated times or transfection with SCR or miR-342. Results are mean ± SD of three independent experiments. *p < 0.05, **p < 0.01; ANOVA followed by Sidak’s multiple comparison test.

**Fig. 4:**
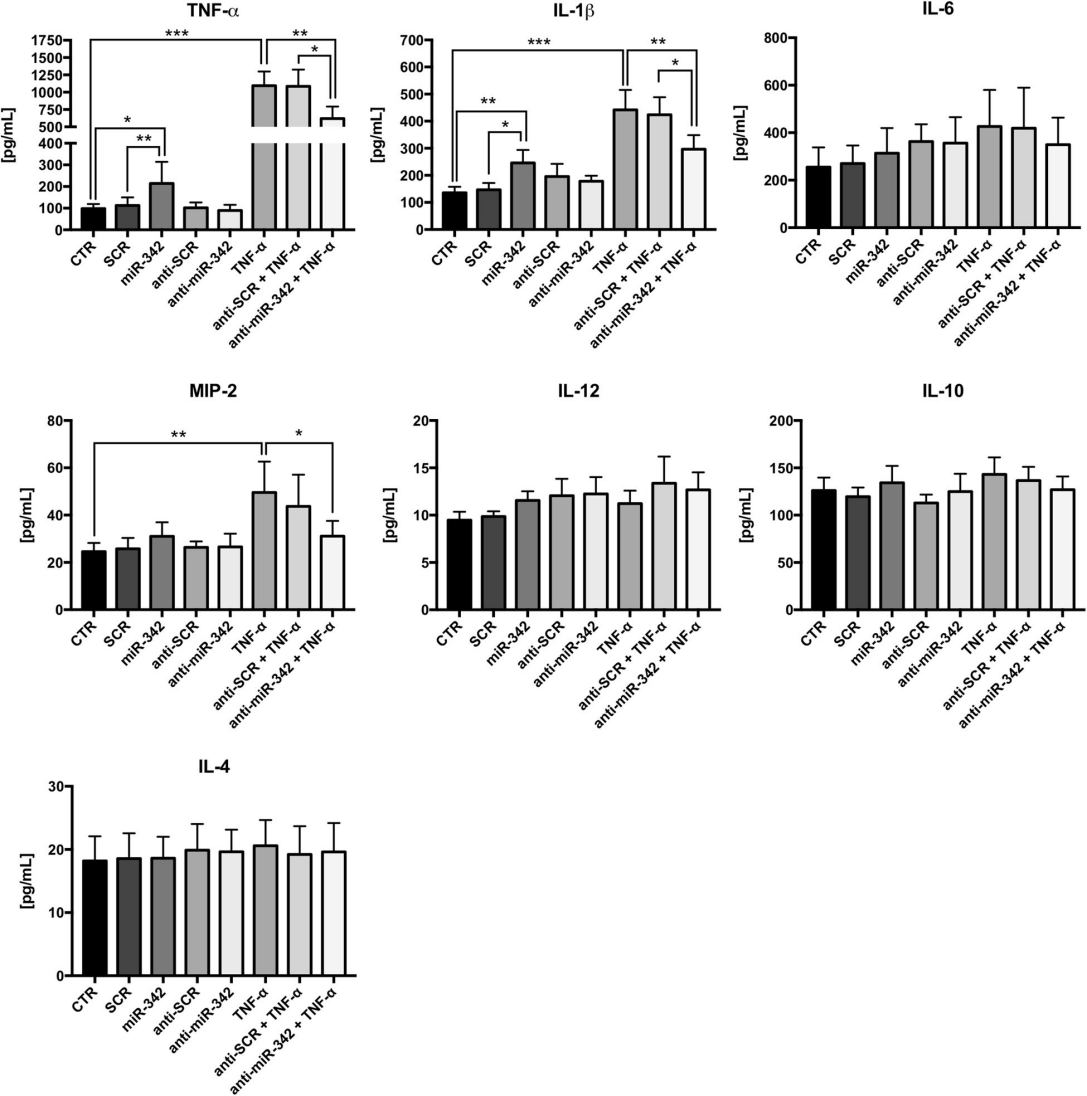
miR-342 impacts cytokine secretion levels in microglia. N9 microglia were transfected with mirVana miRNA mimic/inhibitor mmu-miR-342–3p or mirVana miRNA mimic/inhibitor Negative Control (SCR), using Lipofectamine 2000. When indicated, microglia were also stimulated with TNF-α (20 ng/mL) for 6 h. After that, supernatants were collected and stored at −80 °C. Levels of TNF-α, IL-1β, IL-6, MIP-2, IL-12, IL-10, and IL-4 were quantified by ELISA (mean ± SD, *n* = 5–7). Cytokine concentration was obtained using corresponding standard curve, according to manufacturer’s instructions. Statistical significance: *p < 0.05, **p < 0.01, and ***p < 0.001; Friedman test followed by Dunn’s multiple comparisons test.

To investigate the role of miR-342 in microglia activation following TNF-α stimulation, gain-of-function experiments were performed using the N9 microglia cell line (Supplementary Figure 4). Strikingly, we found that miR-342 overexpression per se, without TNF-α stimulation, was sufficient to increase ph-NF-kB p65 levels compared with non-stimulated (*p* = 0.004) and SCR-transfected microglia cells (*p* = 0.012; Fig. 3a). On the other hand, anti-miR-342 transfection resulted in a decrease in ph-NF-kB p65 levels compared with anti-SCR (*p* = 0.028), but not CTR (Fig. 3a). However, following TNF-α activation, miR-342 inhibition had a significant impact on the reduction of NF-kB p65 phosphorilation levels (vs TNF-α, *p* = 0.044; vs TNF-α + anti-SCR, *p* = 0.037). The translocation of NF-kB to the nucleus under miR-342 overexpression was analyzed using ImageStreamX. Figure 3B shows a representative histogram of the similarity coefficient between NF-kB and nuclei of N9 microglia cells transfected with SCR (green) or miR-342 (yellow), and representative images of a cell with NF-kB in the cytoplasm (non-translocated) or colocalized with the nucleus (translocated). Transient transfection of N9 microglia cells with miR-342 resulted in a clear shift towards a higher similarity coefficient, which indicates that compared with SCR, miR-342 overexpression promotes NF-kB translocation to the nucleus. Quantification of the percentage of cells with nuclear localization of NF-kB shows a significant NF-kB nuclear translocation upon miR-342 overexpression (Fig. 3b). Although not significantly, the treatment with TNF-α also shows a tendency for inducing the translocation of NF-kB into the nucleus (Fig. 3b, 10 min). The inflammatory role of miR-342 was further confirmed by the levels of pro-inflammatory cytokines produced by transfected microglia. Specifically, microglia overexpressing miR-342 show increased levels of TNF-α and IL-1β, compared with non-stimulated (TNF-α, *p* = 0.021; IL-1β, *p* = 0.008) and SCR-transfected microglia cells (TNF-α, *p* = 0.004; IL-1β, *p* = 0.022; Fig. 4). Conversely, miR-342 overexpression had no effect on IL-6, MIP-2, IL-12, IL-10, and IL-4. miR-342 inhibition, without TNF-α stimulation, had no impact on cytokine production compared with anti-SCR control (Fig. 4). In agreement with the RT-qPCR results shown in Fig. 1a, exposure to TNF-α induced the secretion of TNF-α (*p* = 0.0006) and IL-1β (*p* < 0.0001), and also of MIP-2 (*p* = 0.019) compared with control microglia cells (Fig. 4), whereas no differences were detected for IL-6, IL-12, IL-10, and IL-4 (Fig. 4). Importantly, the increased secretion of TNF-α, IL-1β and MIP-2 after TNF-α stimulation, was reduced by miR-342 inhibition (TNF-α, *p* = 0.004; IL-1β, *p* = 0.004; MIP-2, *p* = 0.027, Fig. 4), which supports the hypothesis that miR-342 is needed for TNF-α-driven microglia activation.

### miR-342 promotes NF-kB activation by inhibiting BAG-1

**Fig. 5:**
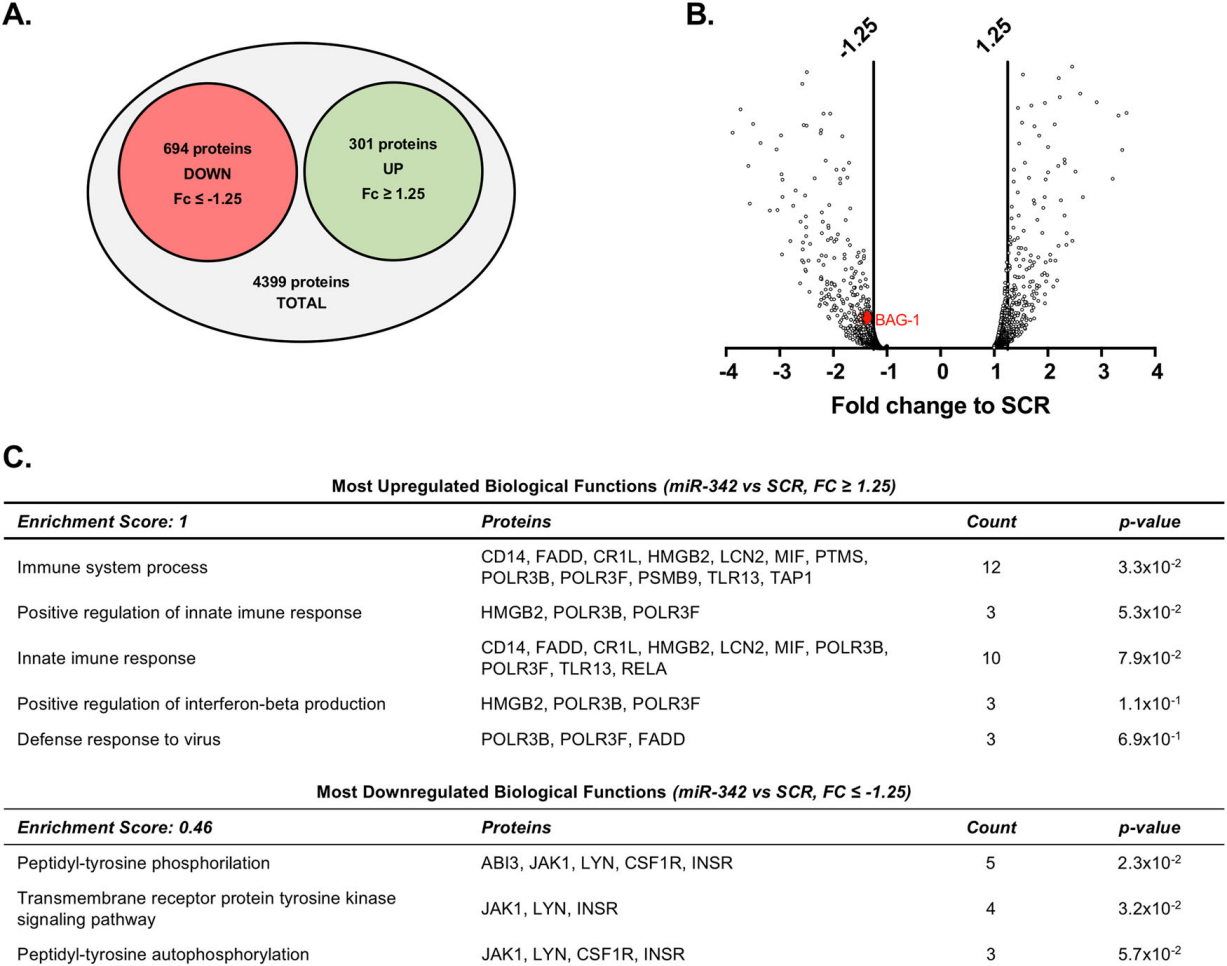
Protein expression profile of miR-342-transfected microglia. Protein identification and quantitation was performed by nano LC-MS/MS. Diagram **a** and volcano plot **b** represent the most differently expressed proteins within all identified proteins (−1.25 ≤ FC to SCR ≥ 1.25, *n* = 2). **c** Most up and downregulated biological functions based on protein expression fold change between miR-342 and SCR. Analysis was performed with DAVID using Functional Annotation Tool. The entire list proteins detected was used as background. “Enrichment score” indicates the biological relevance of the group of proteins involved in the respective set of biological functions, based on the *p* values of all enriched annotation terms. “Count” indicates the number of dysregulated proteins involved in that specific biological function. Full protein names can be found in Supplementary Table 4.

**Fig. 6:**
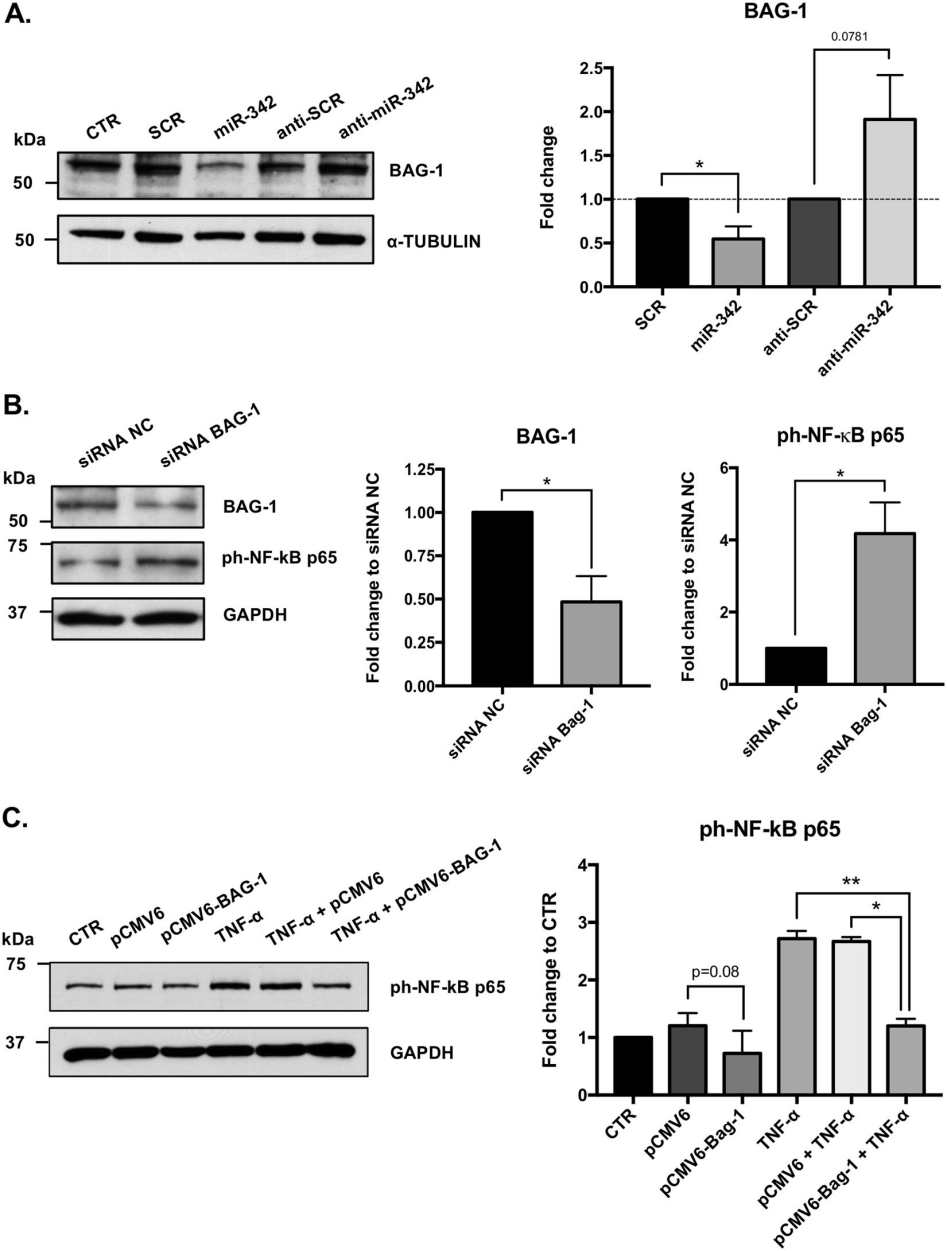
miR-342 induces NF-kB activation by inhibiting BAG-1. **a** BAG-1 expression after miR-342 overexpression/inhibition was addressed by western blot. Results were normalized with α-tubulin and compared with the respective controls (mean ± SD, *n* = 6). To evaluate the involvement of BAG-1 on NF-kB activation, N9 microglia were transfected with a siRNA to silence **b** or with a plasmid (1ug/mL) to overexpress BAG-1 **c**. BAG-1 and ph-NF-kB p65 expression levels were evaluated by western blot. Results were normalized with GAPDH and compared with the respective controls (mean ± SD, *n* = 2–4). Statistical significance: *p < 0.05, Wilcoxon matched-pairs test.

Next, we searched for miR-342 target molecules and pathways/functions that could be involved in the TNF-α-driven microglia activation. Thus, the protein expression profile of miR-342-transfected N9 microglia cells was analyzed (Fig. 5a, b). The nano LC-MS/MS identified 4399 proteins, of which 694 were downregulated (FC to SCR < −1.25) and 301 upregulated (FC > 1.25; Fig. 5a, b and Supplementary Table 4). Interestingly, DAVID functional annotation^28^, revealed that the group of upregulated proteins with the highest biological function enrichment score, are mainly involved in inflammatory responses (Fig. 5c). In the group of most downregulated proteins, we identified a potential candidate, BAG-1 (FC = −1.42; Fig. 5b). BAG-1 was previously described to degrade NF-kB p65, inhibiting inflammatory signaling in dendritic cells^29^. To validate these results, we carried out western blots against BAG-1 on proteins extracts from miR-342 and anti-miR-342 transfected N9 microglial cells. Results show that miR-342 overexpression significantly inhibits BAG-1 expression (mean FC to SCR = 0.546, *p* = 0.031; Fig. 6a), whereas miR-342 inhibition induces an increase on BAG-1 expression (mean FC to anti-SCR = 1.911, *p* = 0.078; Fig. 6a). To confirm if NF-kB activation on miR-342-overexpressing N9 microglia cells occurs via BAG-1 inhibition, we used a siRNA targeting BAG-1 to inhibit its expression. Results clearly show that NF-kB p65 activation is dependent on BAG-1 expression, as its downregulation induced a significant overexpression of ph-NF-kB p65 (mean FC to siRNA NC = 4.18, *p* = 0.012; Fig. 6b). To further explore the role of BAG-1 on NF-kB p65 regulation, we transiently transfected N9 microglia cells with a BAG-1 mammalian expression vector. Although not significant, BAG-1 overexpression showed a trend towards a slight inhibition of NF-kB p65 phosphorylation levels compared with control (vs pCMV6*, p* = 0.08). Nonetheless, it significantly inhibited the upregulation of ph-NF-kB induced by TNF-α (vs TNF-α, *p* = 0.008; vs TNF-α + pCMV6, *p* = 0.014, Fig. 6c). These results show that miR-342 promotes NF-kB activation by inhibiting BAG-1.

### miR-342 overexpression in microglia induces neurotoxicity

After identifying the mechanism by which miR-342 participates on microglia activation, we addressed its functional consequences, specifically in the cross-talk with neurons.

**Fig. 7:**
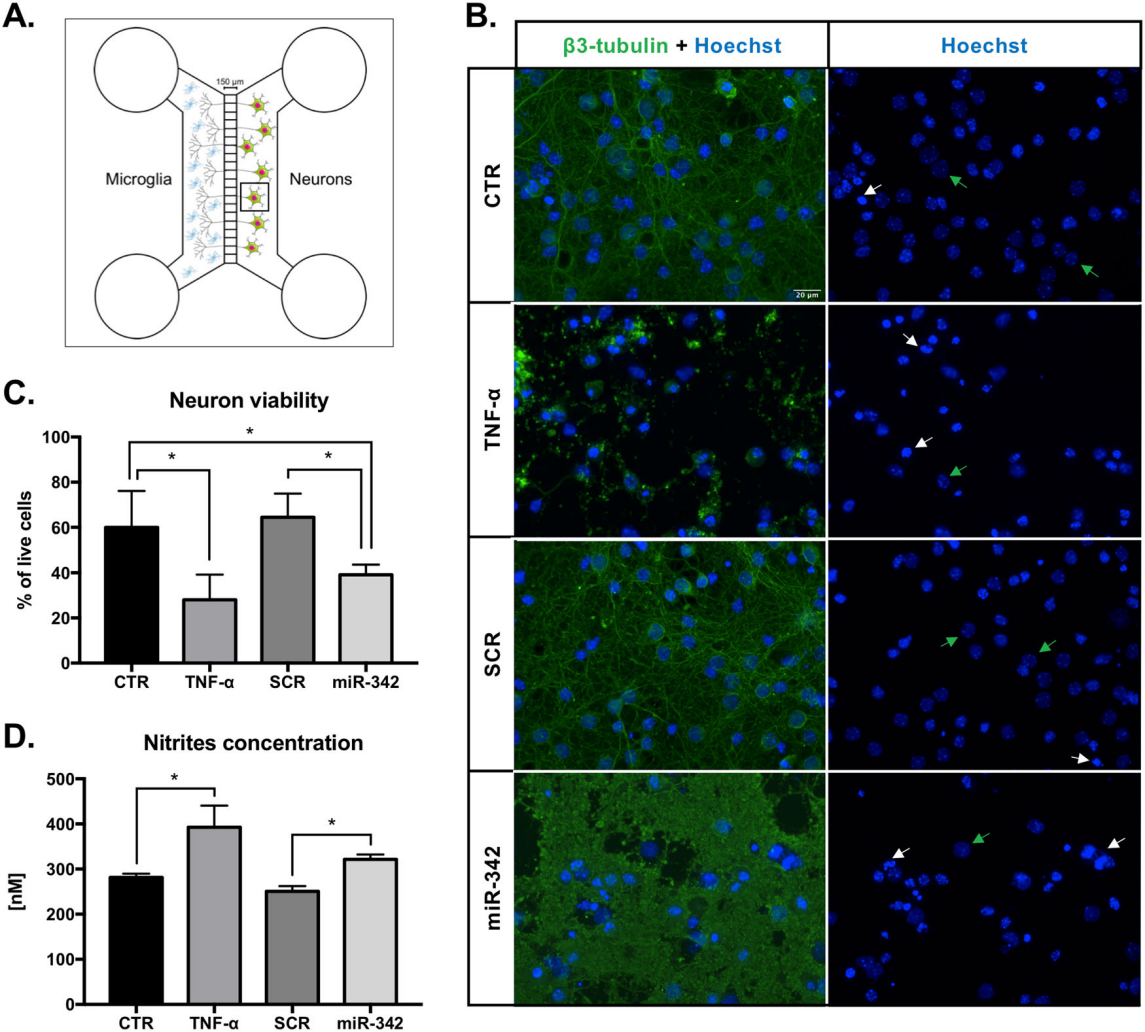
miR-342 overexpression in microglia induces neurotoxicity. **a** Neurons were cultured in PDL-coated coverslips previously attached to the Axon Investigation System. At day 13 of neuronal culture, transfected, TNF-α, or non-stimulated N9 microglia were added to the respective system, in direct contact with axons for 24 h. **b** Immufluorescence images of neurons after co-culture with N9 microglia. Left panel shows neurons stained with anti-β3-tubulin (anti-Alexa 488, green) and Hoechst (blue). Right panel shows only Hoechst nuclear staining used for neuron viability evaluation. White arrows highlight the nucleus of dead neurons, whereas green arrows highlight the nucleus of healthy neurons. Scale bar: 20 µm. **c** Neuron viability was addressed after counting the number of living and dead cells of 10 images per condition (mean ± SD, *n* = 4). **d** Co-cultures’ supernatants were collected for nitrite levels quantification using Griess reagent (mean ± SD, *n* = 4). Statistical significance: *p < 0.05 and **p < 0.01, Friedman test followed by Dunn’s multiple comparisons test.

Primary mouse hippocampal neurons were cultured on the right side of microfluidic chambers for 13 days, allowing axon projection through the device’s microgrooves. Then, transfected/TNF-α-stimulated N9 microglia cells were plated on the left side of the system (Fig. 7a and Supplementary Figure 5). After 24 h of co-culture, the supernatants were collected, and the cells were immunostained for the assessment of neuronal viability. Interestingly, TNF-α-activated and miR-342-transfected microglia had a significant impact on neurons integrity (Fig. 7b). TNF-α stimulated microglia significantly reduced neuron viability by 55%, compared with non-stimulated microglia (FC to CTR = 0.45, *p* = 0.028; Fig. 7c). miR-342-overexpressing N9 microglia cells also significantly induced neurotoxicity, reducing cell viability by 33% compared with CTR (FC to CTR = 0.67, *p* = 0.041, Fig. 7c) and in 38% compared with SCR-transfected microglia (FC to CTR = 0.62, *p* = 0.026, Fig. 7c).

**Fig. 8:**
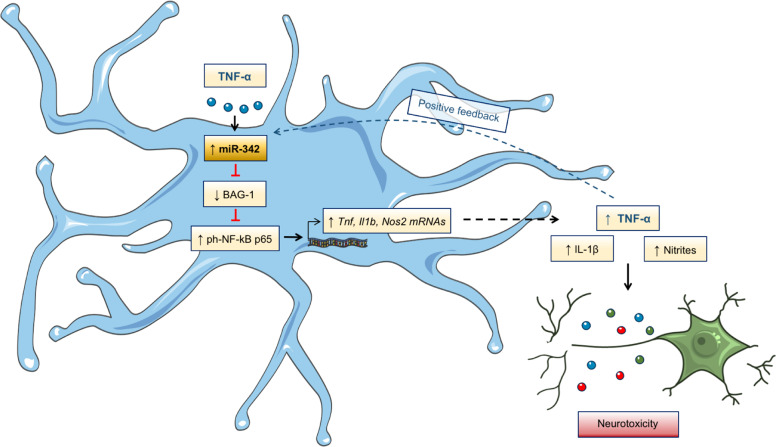
Graphical abstract–TNF-alpha-induced miR-342 promotes microglia activation through NF-kB and induces neurotoxicity. We found miR-342 to be upregulated in microglia activated with TNF-α. miR-342 promotes NF-kB activation by inhibiting BAG-1, leading to the overexpression of pro-inflammatory mediators, including TNF-α, in a positive feedback loop, possibly perpetuating microglia activation. Importantly, inhibition of miR-342 attenuated TNF-α-driven microglia activation. Moreover, microglia activation by miR-342 led to increased neurotoxicity with high levels of nitrites being detected in co-cultures supernatants.

Considering that overactivated microglia increases the production of pro-inflammatory cytokines and nitric oxide species, which may have deleterious effect to the surrounding cells, we next determined nitrites concentration in the co-culture supernatants. In fact, the quantification shows a significant increase in the levels of nitrites in the supernatants of both TNF-α-stimulated (*p* = 0.0437) and miR-342-overexpressing (*p* = 0.0437) microglia co-cultures (Fig. 7d). These results support our hypothesis that miR-342-mediated microglia activation impacts the cross-talk with neurons(Fig. 8).

## Discussion

Microglia are the primary immune effector cells in the CNS. Impairment of their normal structure or function, caused by either inflammatory activation (e.g., following infection, trauma, autoimmune, or neurodegenerative diseases) or by their decline and senescence (e.g., during aging or Alzheimer’s disease), may cause damage in neuronal function and neurogenesis^30–33^. In addition, systemic alterations in the cytokine profile have been associated with distinct neurological and psychiatric diseases (e.g., depression, bipolar disorder, or schizophrenia)^30,34,35^. Pro-inflammatory cytokines, such as TNF-α, a central mediator of neuroinflammation^36^, have access to the brain through humoral and neural routes, leading to microglia activation^37^. In this work, we explored the impact of TNF-α on miRNAs expression in microglia and the mechanisms by which these miRNAs regulate cell activation. We show, for the first time, that miR-342 is a crucial mediator of TNF-α-driven microglia activation.

miR-342 was found upregulated in primary rat microglia activated with TNF-α. To explore the role of miR-342 on TNF-α-driven microglia activation we used a mouse cell line (N9), owing to the low (non-viral) transfection efficiency when working with primary microglia cells. Moreover, of the few microglia cell lines available, N9 is the one of the most commonly used and widely accepted. Importantly, the miR-342–3p sequence shows 100% homology between rat and mouse (Supplementary Figure 6).

Following miR-342 overexpression, we found that miR-342 per se, was sufficient to activate the NF-kB pathway, as shown by the increased ph-NF-kB p65 levels and NF-kB p65 nuclei translocation. Notably, miR-342-driven NF-kB p65 activation led to increased secretion of TNF-α and IL-1β. Conversely, miR-342 inhibition strongly decreased the levels of these cytokines after TNF-α stimulation, suggesting that this miRNA is needed for microglia activation. Moreover, we observed that TNF-α mRNA and secreted levels were upregulated after TNF-α stimulation, in a positive feedback loop, possibly perpetuating neuroinflammation and ultimately promoting brain pathological conditions^13^. In line with our results, Kuno et al.^13^ proposed the microglia-derived TNF-α autocrine activation occurs via TNFR1 signaling pathway.

miRNAs act by degradation or translation inhibition of their target mRNAs^16^. Therefore, after observing that miR-342 overexpression increases the levels of ph-NF-kB p65, we hypothesized that this miRNA could be repressing an inhibitor of NF-kB. Consequently, we screened the LC-MS/MS data for proteins that were significantly downregulated and previously described as repressors of NF-kB. BAG-1 was the only candidate found to meet both criteria^29^ (Supplementary Table 4). BAG-1 is known to interact with heat shock protein 70 (Hsp70) family of molecular chaperones, displaying a variety of cytoprotective activities and effects on signal transduction and transcription, suggesting a function in overcoming cellular stress and inflammation^38,39^. Tanaka et al.^29^ described that BAG-1, when associated with HSP70, can promote NF-kB p65 degradation in LPS-treated dendritic cells. The authors reported that mouse dendritic cells deficient in either HSP70 or BAG-1 had more nuclear p65 and produced more pro-inflammatory cytokines than did wild-type dendritic cells^29^, implicating BAG-1 as a negative regulator of pro-inflammatory NF-kB signaling in dendritic cells. Our study shows, for the first time, that BAG-1 is a mediator of NF-kB signaling in microglia, and that BAG-1 protein levels are regulated by miR-342.

Nevertheless, it is important to note that miRNAs can target multiple transcripts, as such we do not exclude that other proteins with relevance for microglia activation, can also be affected by miR-342 overexpression. Specifically, DAVID functional annotation analysis revealed that inflammation-related processes were the most upregulated biological functions associated with miR-342 overexpression (Fig. 5c). Particularly, FADD and MIF expression regulation by miR-342 should be further explored, as they are known to potentiate NF-kB activation and pro-inflammatory cytokines production^40,41^.

The deleterious effects of excessive production of cytokines and nitric oxide species in neurons have been extensively studied^32,37^. For instance, TNF-α can potentiate glutamate-mediated cytotoxicity by two complementary mechanisms: indirectly, by inhibiting glutamate transport on astrocytes, and directly, by stimulating extensive microglial and/or astrocyte glutamate release in an autocrine manner. This disturbs the balance of excitation and inhibition, resulting in a higher synaptic excitatory/inhibitory ratio—excitoneurotoxicity^37,42,43^. On the other hand, microglial derived nitric oxide inhibits neuronal respiration, resulting in glutamate release and subsequent excitotoxicity^44,45^. As such, to address the effect of miR-342 overexpression in the cross-talk with neurons, we co-cultured microglia with hippocampal neurons. Interestingly, we found that, in both TNF-α stimulated and miR-342 transfected microglia, neuron viability was drastically affected compared with the controls. We hypothesize that this may be owing to an increased production of the pro-inflammatory cytokines IL-1β and TNF-α (Results section 3), but also owing to increased levels of nitrites detected in the supernatants of these co-cultures. Not surprisingly, and given the upregulation of *Nos2*, supernatants of TNF-α stimulated microglia show an increased level of nitrites. Moreover, miR-342 overexpression also induced neuronal cell death, revealing that miR-342 impacts microglia activation as well as its cross-talk with neurons. In the current work, the possibility that these findings are cell line specific cannot be completely ruled out. It would be interesting to pursue this work in primary microglia cells, and further to perform in vivo administration of miR-342 targeted to microglia, to clarify its impact on the cross-talk between microglia and neurons, as well as the repercussion on animals’ behavior, particularly in animal models of neuroinflammation.

These findings support miR-342 as potential target to resolve neuroinflammation, characterized by increased levels of TNF-α, sustained microglia activation, and often associated with the development of neurological and psychiatric disorders.

## Supplementary information

Supplementary Figures and Tables Legends

Supplementary Figure 1

Supplementary Figure 2

Supplementary Figure 3

Supplementary Figure 4

Supplementary Figure 5

Supplementary Figure 6

Supplementary Table 1

Supplementary Table 2

Supplementary Table 3

Supplementary Table 4
