# Supplementary material for: TNF-alpha-induced microglia activation requires miR-342: impact on NF-kB signaling and neurotoxicity
Source: Cell Death Dis. 2020 Jun 2;11(6):415. doi: 10.1038/s41419-020-2626-6 (PMC7265562; doi:10.1038/s41419-020-2626-6)
Supplement: Supplementary file 1 — Supplementary Figures and Tables Legends [file 41419_2020_2626_MOESM1_ESM.docx]

**Supplementary Figure and Table Legends**

**Supplementary Figure 1. Rat microglia isolation and *in vitro* stimulation procedures.** (i) Newborn rat pups (P1-P2) were decapitated and brains separated from the skull; (ii) Cerebellum and meninges were carefully removed for efficient brain dissection. Dissected tissue was treated with DNase and trypsin before being dissociated and plated; (iii) Mixed glial cells (astrocytes, oligodendrocytes and microglia) were cultured with 10% FBS supplemented media in T75 poly D-lysine coated flasks for 21 days; (iv) Microglia were obtained at day 14 and 21 of culture by shaking the flasks at 37°C for 2h at 150 rpm; (v) Isolated microglia were re-seeded in 6-well plates, allowed to adhere for 48h and then stimulated for 6h with TNF-α (20 ng/mL) or LPS (100 ng/mL); (vi) Activated microglia were finally harvested for RNA or protein extraction.

**Supplementary Figure 2. Microglia characterization by flow cytometry.** (A) Microscopy image of rat microglia, 48h after being isolated from mixed glial culture and re-seeded (scale bar, 100 µm). Dot plots and histograms represent microglia surface expression evaluation by flow cytometry.

**Supplementary Figure 3. Rat microglia ph-NF-kB p65 expression after LPS and TNF-α stimulation.** Rat microglia were obtained from mixed glial cultures. Isolated microglia were re-seeded in 6-well plates, allowed to adhere for 48h and then stimulated for 6h, 12h, 24h and 48h with LPS (100 ng/mL) or TNF-α (20 ng/mL).

**Supplementary Figure 4. Transfection efficiency of miRVana mmu-miR-342-3p mimics in N9 microglia cells.** Transfection efficiency was evaluated by RT-qPCR and compared with the controls (n=2). Statistical significance: ***p* < 0.001.

**Supplementary Figure 5. Hippocampal Neurons-N9 microglia co-culture in the Axon Investigation System.** (A) N9 microglia cells stained with anti-IBA1 (anti-Alexa 594, red). (B) Left side of the device containing N9-microglia in direct contact with axons. (C) Right side of the device containing neuron cell bodies stained with anti-β3-tubulin (anti-Alexa 488, green). (D) Axon Investigation System microgrooves (150 µm length) through which neurons project their axons and communicate with microglia. Cell nuclei were stained with Hoechst (Blue).

**Supplementary Figure 6. miR-342-3p sequences in mouse and rat.** miR-342-3p sequences for mouse and rat were obtained in miRbase ([www.mirbase.org](http://www.mirbase.org)).

**Supplementary Table 1.** **Oligonucleotides sequences for RT-qPCR.** Oligonucleotides used to amplify rat mRNAs encoding inflammatory markers and rat *Gapdh* used as a reference gene, based on GenBank sequences. Abbreviations: Nos2, nitric oxide synthase 2; Il6, interleukin 6; Tnf, tumor necrosis factor; Il1b, interleukin 1 beta; Msr1, macrophage scavenger receptor 1; Il10, Interleukin 10 macrophage; Gapdh, glyceraldehyde 3-phosphate dehydrogenase.

**Supplementary Table 2.** **Antibodies used for protein detection**. Antibodies were diluted following manufacturer’s instructions in 5% BSA in TBS-T 0.1% or 5% Milk in TBS-T 0.1%, for detection of phosphorylated or total forms, respectively. Abbreviations: NF-kB p65, Nuclear factor kappa B subunit p65; BAG-1, BAG family molecular chaperone regulator 1; COX-2, cyclooxygenase-2.

**Supplementary Table 3.** **miRNA microarray results.** Most up- and downregulated miRNAs in TNF-α *versus* non-stimulated (CTR) primary rat microglia.

**Supplementary Table 4.** **Most up- and downregulated proteins in miR-342 *versus* SCR transfected N9 microglia.** Protein identification and quantitation was performed by nano LC-MS/MS. List contains the most up (FC > 1.25, blue) and downregulated (FC < 0.8/-1.25, green) proteins based on expression fold change between miR-342 and SCR (adjusted p-value < 0.05).
