# Supplementary figures and images for: TNF-alpha-induced microglia activation requires miR-342: impact on NF-kB signaling and neurotoxicity
Source: Cell Death Dis. 2020 Jun 2;11(6):415. doi: 10.1038/s41419-020-2626-6 (PMC7265562; doi:10.1038/s41419-020-2626-6)

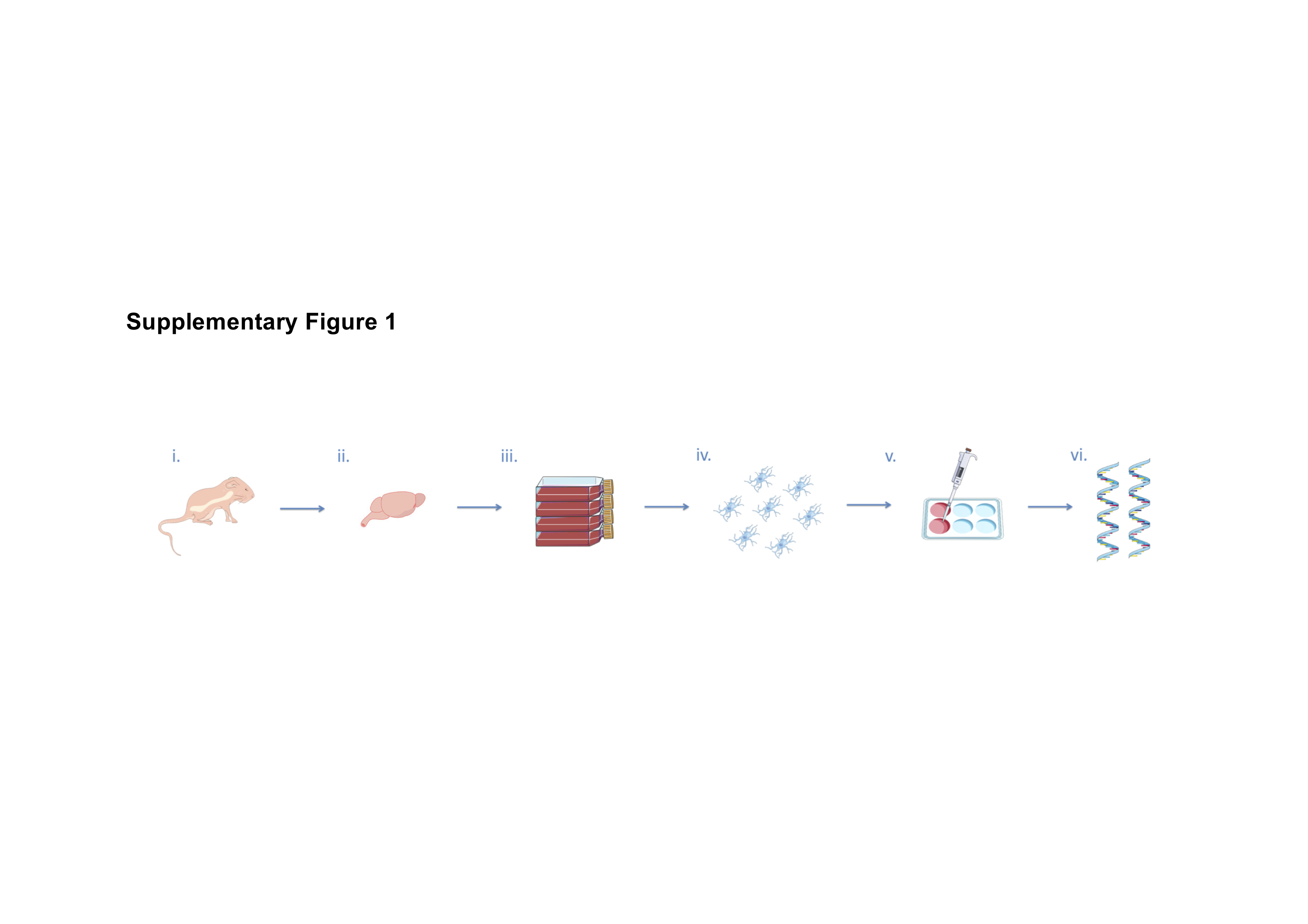

Supplement: Supplementary file 2 — Supplementary Figure 1 [file 41419_2020_2626_MOESM2_ESM.tif]

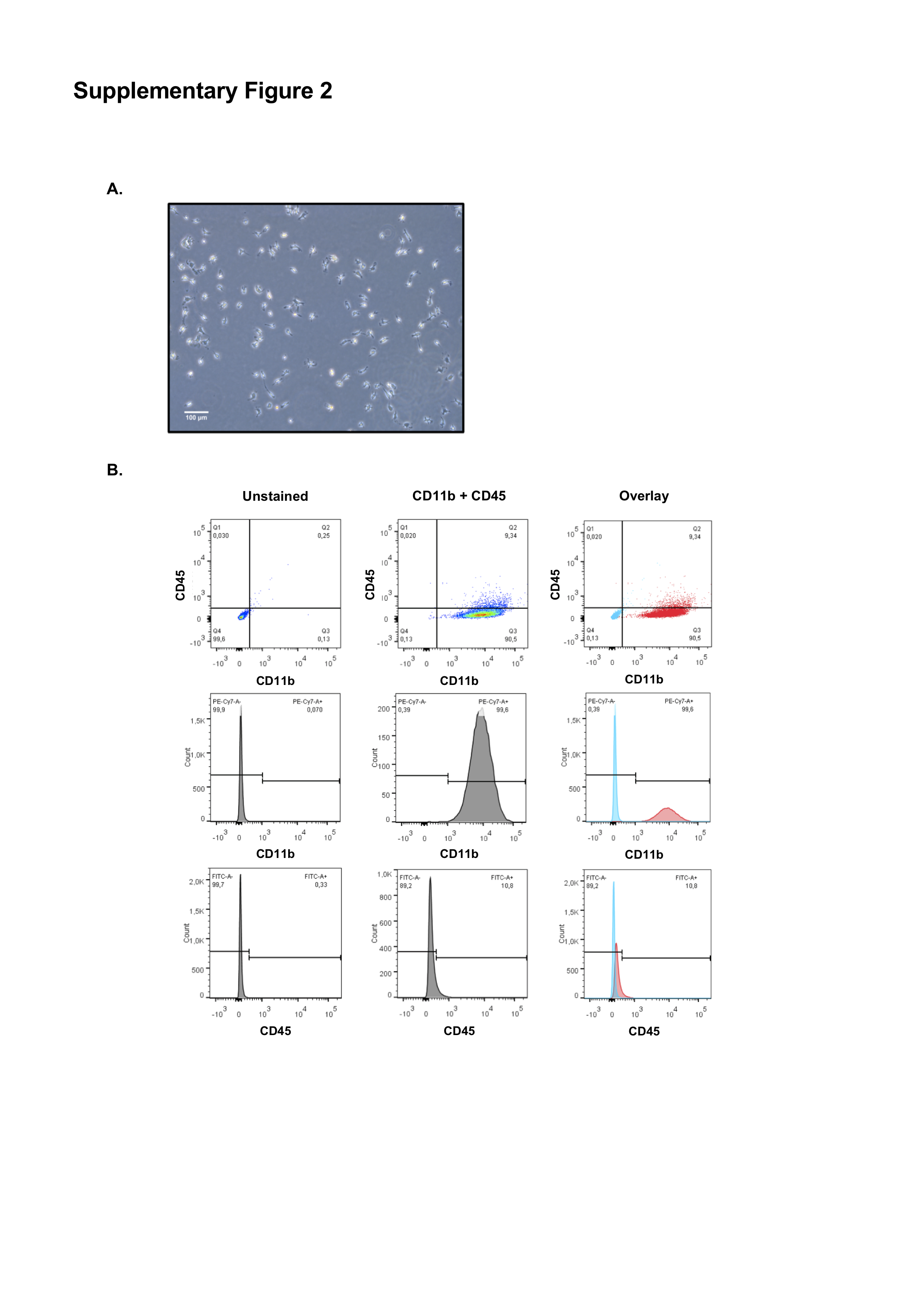

Supplement: Supplementary file 3 — Supplementary Figure 2 [file 41419_2020_2626_MOESM3_ESM.tif]

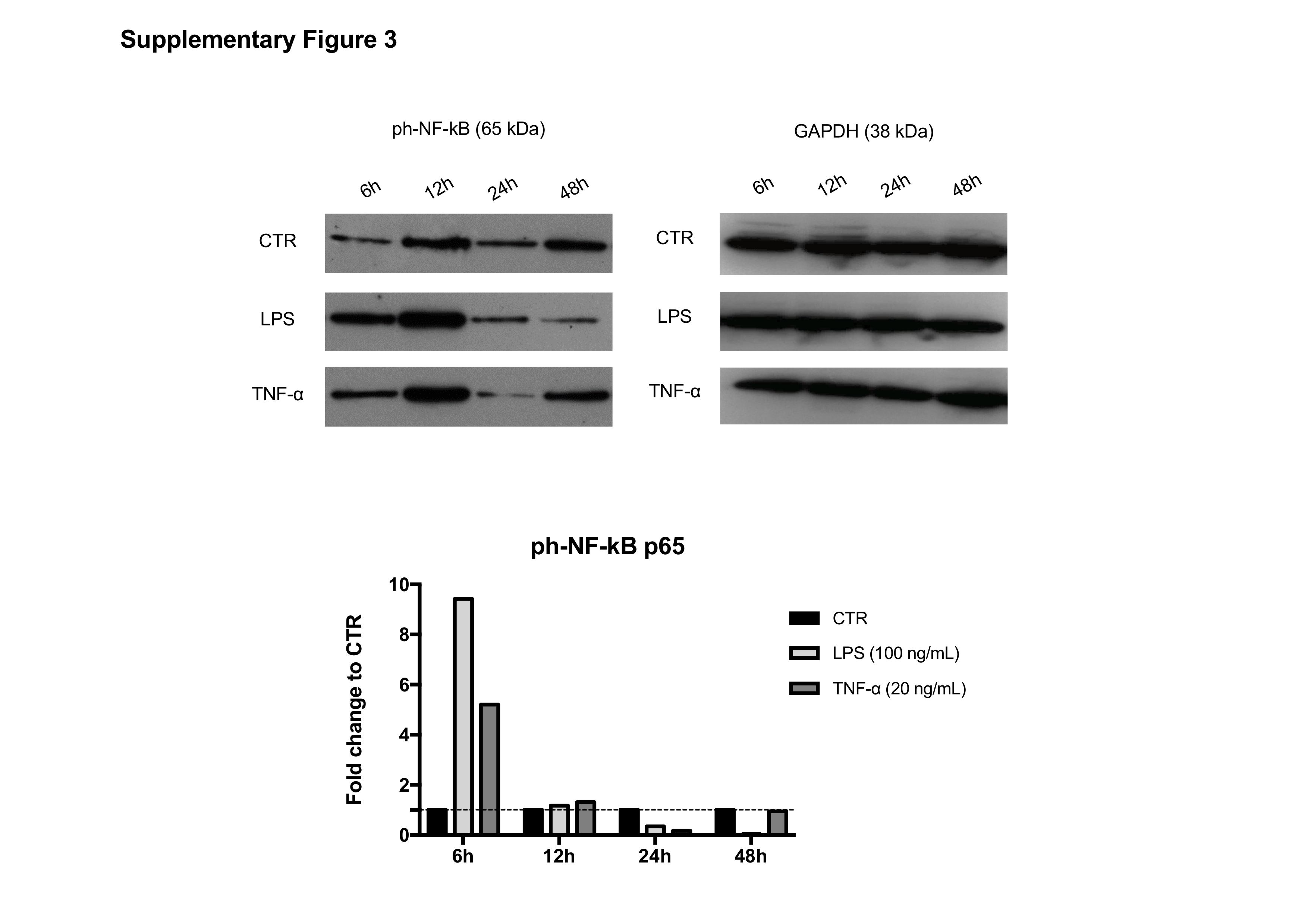

Supplement: Supplementary file 4 — Supplementary Figure 3 [file 41419_2020_2626_MOESM4_ESM.tif]

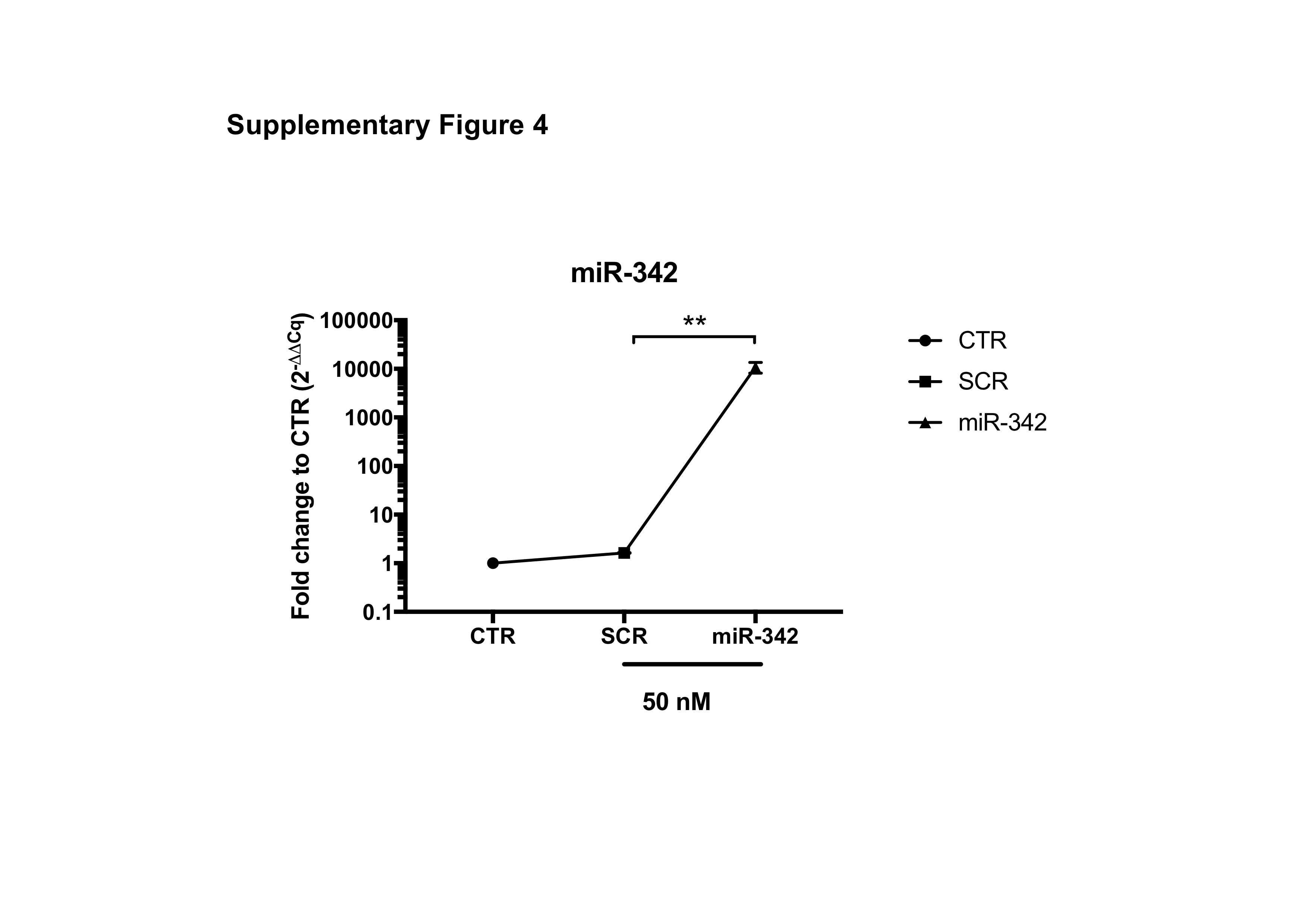

Supplement: Supplementary file 5 — Supplementary Figure 4 [file 41419_2020_2626_MOESM5_ESM.tif]

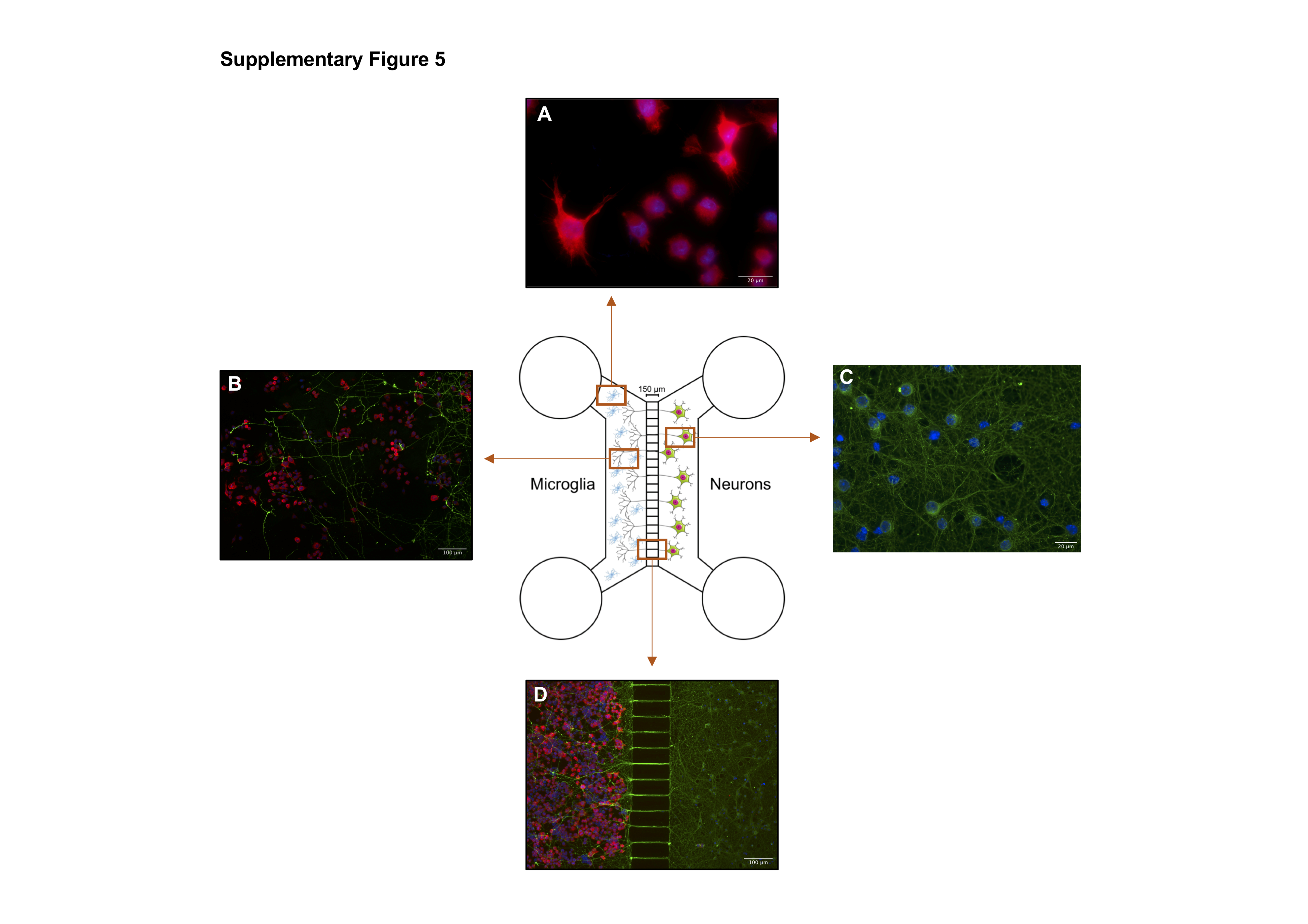

Supplement: Supplementary file 6 — Supplementary Figure 5 [file 41419_2020_2626_MOESM6_ESM.tif]

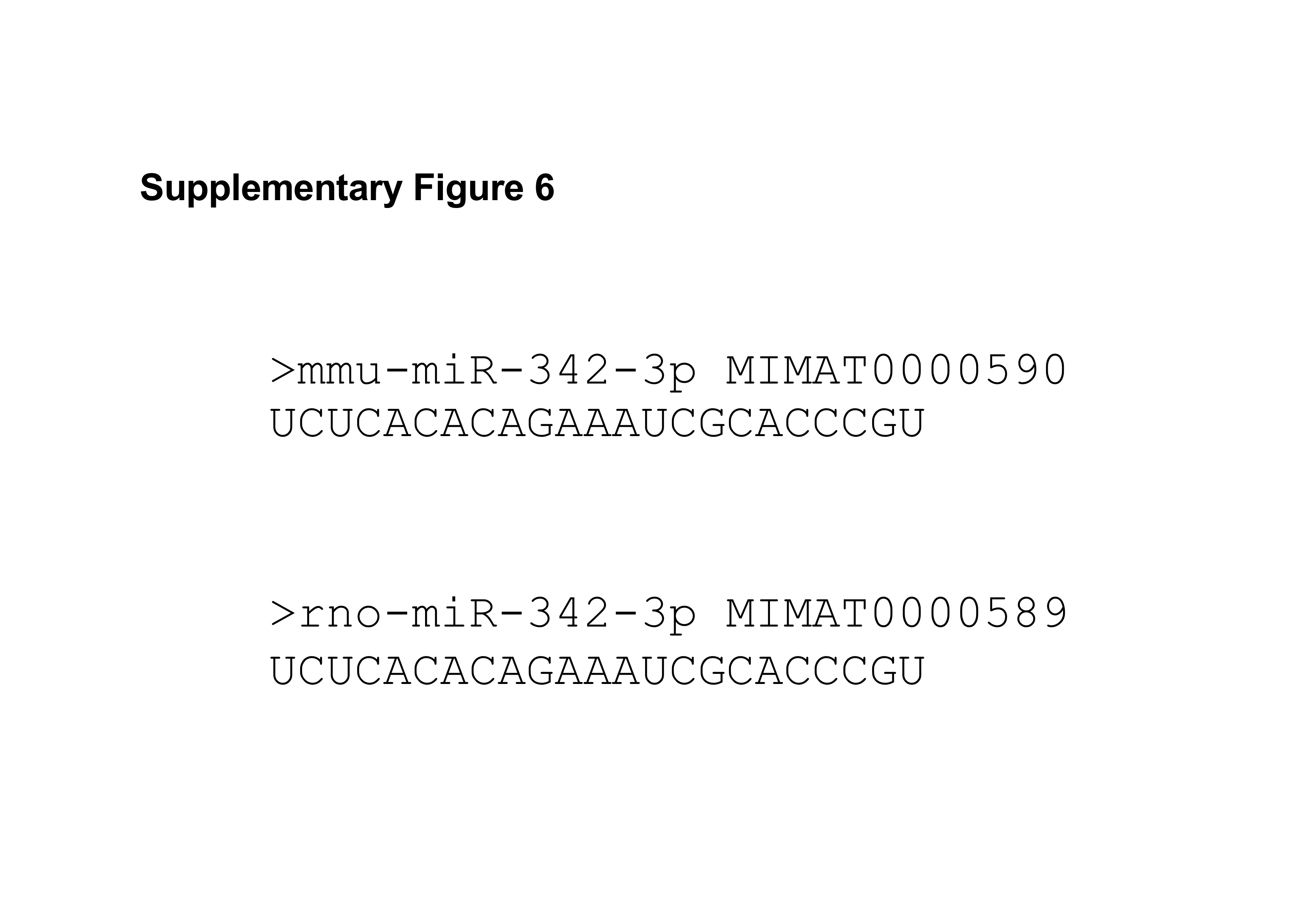

Supplement: Supplementary file 7 — Supplementary Figure 6 [file 41419_2020_2626_MOESM7_ESM.tif]

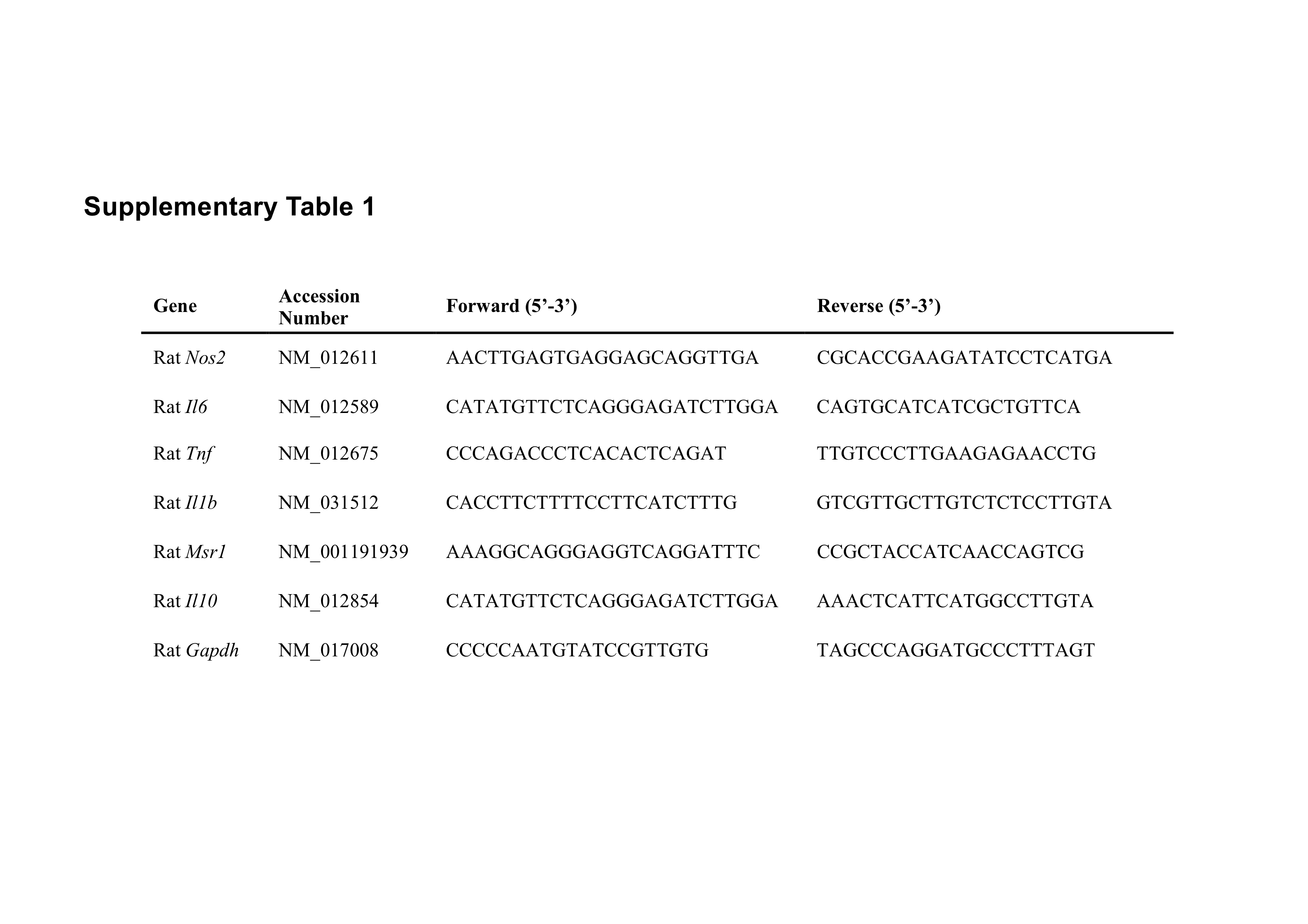

Supplement: Supplementary file 8 — Supplementary Table 1 [file 41419_2020_2626_MOESM8_ESM.tif]

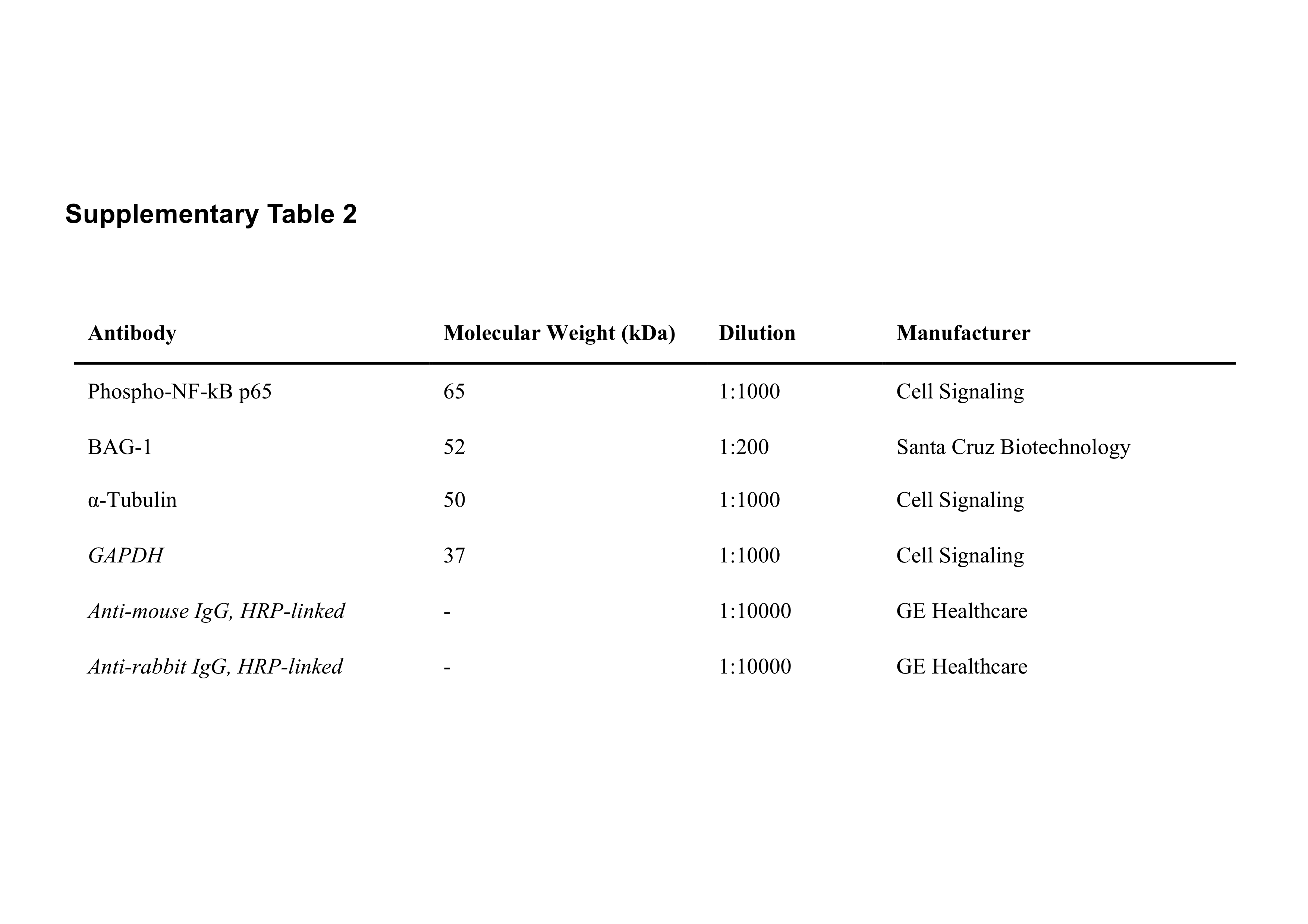

Supplement: Supplementary file 9 — Supplementary Table 2 [file 41419_2020_2626_MOESM9_ESM.tif]

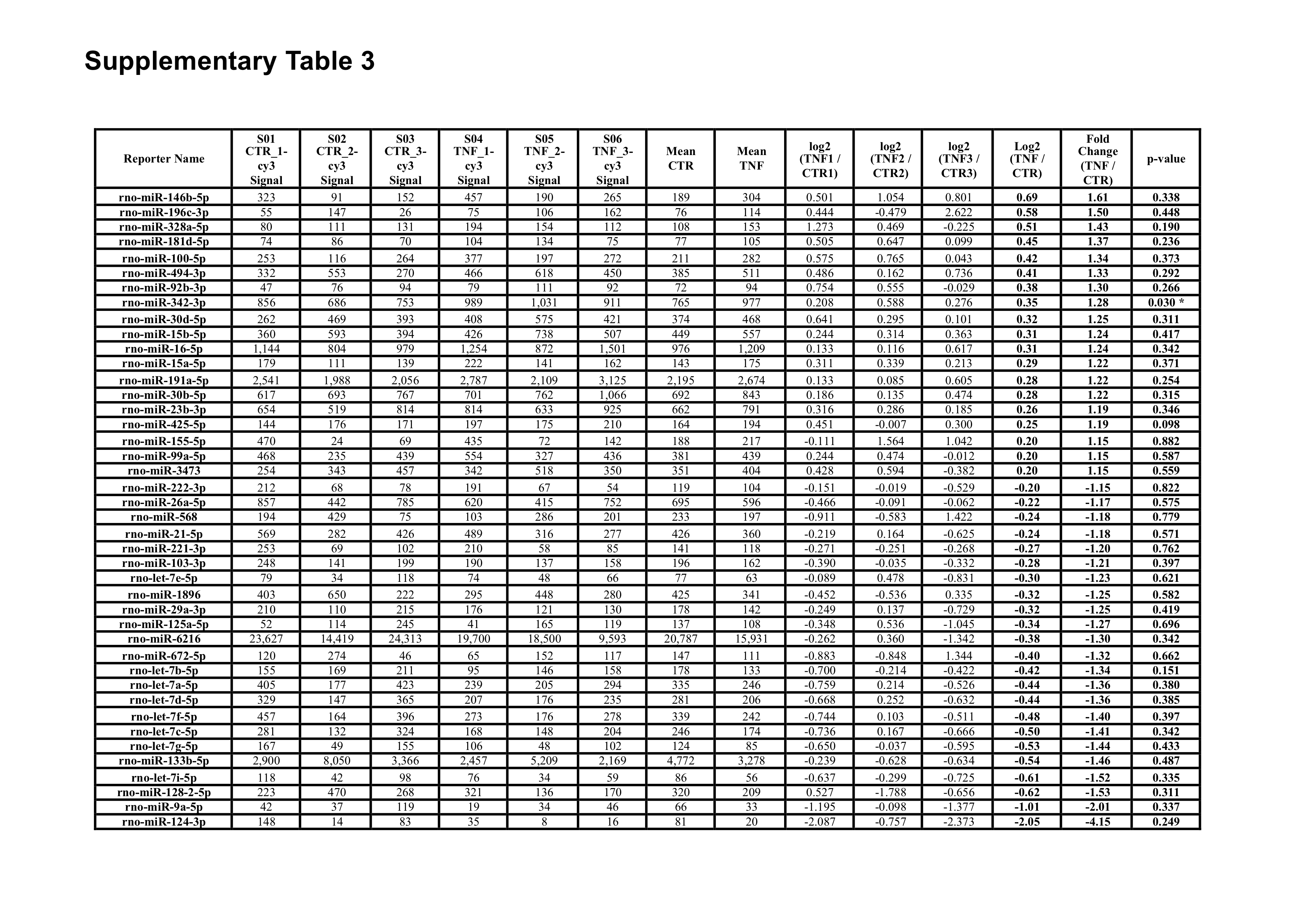

Supplement: Supplementary file 10 — Supplementary Table 3 [file 41419_2020_2626_MOESM10_ESM.tif]
