# Supplementary material for: TNF-alpha-induced microglia activation requires miR-342: impact on NF-kB signaling and neurotoxicity
Source: Cell Death Dis. 2020 Jun 2;11(6):415. doi: 10.1038/s41419-020-2626-6 (PMC7265562; doi:10.1038/s41419-020-2626-6)
Supplement: Supplementary file 11 — Supplementary Table 4 [file 41419_2020_2626_MOESM11_ESM.docx]

**Supplementary Table 4**

| **Protein ID** | **Abundance Ratio** | **Abundance Ratio Adj. P-Value** | **Abundances (Grouped)** | **Abundances (Grouped)** | **Protein FDR Confidence** |
| --- | --- | --- | --- | --- | --- |
|  | **miR-342/SCR** | **miR-342/SCR** | **miR-342** | **SCR** | **Combined** |
| UPRT | 23.238 | 5.677E-16 | 4647.5 | 200 | High |
| TAF12 | 22.025 | 5.677E-16 | 4405 | 200 | High |
| CYP4A14 | 12.846 | 5.677E-16 | 2569.1 | 200 | Low |
| ENDOG | 11.472 | 5.677E-16 | 219.7 | 19.1 | High |
| EXTL2 | 10.029 | 1.8015E-12 | 2005.7 | 200 | Medium |
| SRD5A3 | 8.975 | 5.677E-16 | 1795 | 200 | High |
| PIK3CB | 8.611 | 5.677E-16 | 1722.2 | 200 | High |
| MCUR1 | 8.543 | 1.9776E-11 | 1708.6 | 200 | High |
| PTMS | 8.153 | 2.0068E-10 | 1630.6 | 200 | High |
| CST6 | 7.042 | 1.0481E-13 | 1408.4 | 200 | Low |
| DENND6A | 6.939 | 1.8955E-09 | 1387.9 | 200 | High |
| RAPGEF4 | 6.785 | 9.3413E-14 | 191.1 | 28.2 | High |
| K16 | 6.552 | 7.1759E-14 | 889.3 | 75.7 | High |
| PSMB9 | 6.512 | 5.677E-16 | 502.6 | 65 | High |
| DDT | 6.149 | 1.1274E-11 | 306.7 | 49.9 | High |
| SSUH2 | 5.267 | 7.7522E-09 | 1053.4 | 200 | Low |
| PRC1 | 4.746 | 1.6097E-10 | 949.2 | 200 | High |
| CYLD | 4.692 | 3.8631E-07 | 938.4 | 200 | High |
| MCRIP1 | 4.635 | 1.3735E-06 | 430.8 | 92.9 | High |
| UBXN6 | 4.453 | 5.677E-16 | 1503.2 | 94.4 | High |
| AAGAB | 4.356 | 1.4922E-11 | 871.1 | 200 | High |
| CCDC12 | 4.338 | 5.677E-16 | 258 | 59.5 | High |
| ARF5 | 4.276 | 8.0242E-07 | 388.9 | 90.9 | High |
| MAP4 | 4.268 | 2.0835E-08 | 853.5 | 200 | Low |
| LAMC1 | 4.227 | 1.0983E-06 | 845.5 | 200 | Medium |
| TSR3 | 4.195 | 1.8125E-09 | 179.1 | 42.7 | High |
| EEF1D | 3.855 | 1.196E-07 | 770.9 | 200 | High |
| TSC1 | 3.546 | 1.4424E-06 | 709.3 | 200 | High |
| TMCO3 | 3.469 | 7.7416E-05 | 209.5 | 60.4 | High |
| AASDHPPT | 3.388 | 0.00034216 | 183.5 | 54.2 | High |
| KRT42 | 3.384 | 6.8553E-08 | 285.4 | 84.3 | High |
| INO80 | 3.316 | 8.637E-05 | 663.1 | 200 | Medium |
| AKR1B3 | 3.26 | 1.9376E-06 | 652 | 200 | High |
| MTIF2 | 3.212 | 0.00109429 | 642.5 | 200 | High |
| TAF15 | 2.911 | 5.0241E-05 | 216.3 | 74.3 | High |
| SMG9 | 2.655 | 2.5512E-06 | 137.6 | 51.8 | High |
| IST1 | 2.653 | 0.00228357 | 43.3 | 93.8 | High |
| TSC22D2 | 2.617 | 3.0137E-08 | 237.1 | 90.6 | High |
| DBR1 | 2.606 | 3.5712E-05 | 75.3 | 95.8 | High |
| PPT2 | 2.516 | 0.00082386 | 167.9 | 66.7 | High |
| MINDY3 | 2.478 | 3.5852E-06 | 495.6 | 200 | High |
| BCAS3 | 2.457 | 0.01327979 | 491.4 | 200 | High |
| ITIH3 | 2.452 | 1.2147E-05 | 241.8 | 92.1 | High |
| BCLAF1 | 2.426 | 3.3367E-10 | 305.4 | 200 | High |
| TMEM177 | 2.383 | 9.7446E-07 | 476.7 | 200 | High |
| CNOT8 | 2.353 | 0.00972524 | 197.2 | 83.8 | High |
| BRD8 | 2.345 | 0.01565076 | 469 | 200 | High |
| ATOX1 | 2.318 | 0.00058292 | 463.7 | 200 | High |
| H2AW | 2.316 | 5.677E-16 | 243.4 | 99.4 | High |
| GDAP2 | 2.313 | 0.00050354 | 231.2 | 100 | High |
| CETN2 | 2.294 | 1.9055E-09 | 229 | 72.5 | High |
| LRWD1 | 2.264 | 6.5073E-07 | 222.1 | 98.1 | High |
| CR1L | 2.246 | 0.0026032 | 449.2 | 200 | High |
| DDX55 | 2.222 | 4.1026E-05 | 62.3 | 200 | High |
| MYC | 2.203 | 1.9194E-05 | 135.3 | 61.4 | Low |
| ITPR2 | 2.201 | 0.01620303 | 200 | 200 | High |
| COX1 | 2.197 | 0.00065402 | 424.8 | 96.6 | High |
| LACC1 | 2.151 | 6.9844E-06 | 430.2 | 200 | High |
| POGLUT3 | 2.142 | 0.02943154 | 176.2 | 82.2 | High |
| LTV1 | 2.127 | 0.00012275 | 211.6 | 45.2 | High |
| SYNE1 | 2.09 | 6.9598E-06 | 185.1 | 88.6 | Low |
| CRELD1 | 2.05 | 0.02926357 | 410 | 200 | High |
| CRADD | 2.044 | 0.02325839 | 162.1 | 79.3 | High |
| LEMD2 | 2.003 | 0.00030554 | 266.3 | 85.5 | High |
| SETD7 | 2.003 | 0.00084851 | 148.7 | 74.2 | High |
| LHPP | 1.991 | 1.3495E-06 | 257 | 99.2 | High |
| KRT1 | 1.979 | 2.4515E-12 | 165.3 | 97.1 | High |
| MRPL30 | 1.958 | 0.03133431 | 160 | 81.7 | High |
| CERS6 | 1.947 | 5.2039E-05 | 194.7 | 100 | High |
| ACTR5 | 1.943 | 0.00229041 | 143.6 | 99.2 | High |
| TULP3 | 1.939 | 0.04855559 | 139.3 | 71.8 | High |
| KCTD5 | 1.914 | 0.00351456 | 97.2 | 50.8 | High |
| MAPKAP1 | 1.896 | 0.00963082 | 188.3 | 99.3 | Low |
| HIST2H2AB | 1.861 | 9.4659E-09 | 244 | 97.3 | High |
| ZNF207 | 1.841 | 0.0001935 | 154.9 | 76.8 | High |
| ANKS1 | 1.821 | 0.02580484 | 196.1 | 58.1 | High |
| FBXW11 | 1.806 | 0.00572234 | 136.6 | 75.6 | High |
| KRT78 | 1.804 | 1.9488E-10 | 165 | 99.4 | High |
| H3F3C | 1.749 | 0.00013298 | 173.3 | 99 | High |
| PEX1 | 1.742 | 0.03159714 | 124 | 71.2 | High |
| TMTC3 | 1.739 | 0.00260124 | 347.7 | 200 | High |
| CNOT10 | 1.71 | 0.03616002 | 95.1 | 55.6 | High |
| NUDCD3 | 1.695 | 5.7705E-05 | 211.8 | 99.7 | High |
| INTS9 | 1.684 | 0.02390757 | 168.1 | 99.8 | High |
| HMGB2 | 1.678 | 2.711E-08 | 169.4 | 99.4 | High |
| PIEZO1 | 1.671 | 0.02989171 | 334.2 | 200 | High |
| TRMU | 1.654 | 0.03946552 | 89.8 | 97.6 | High |
| APPL1 | 1.649 | 0.00105168 | 156.2 | 97.7 | High |
| KRT73 | 1.636 | 8.5408E-08 | 153.2 | 93.6 | High |
| FTH1 | 1.62 | 4.0934E-06 | 149 | 92.2 | High |
| KRT14 | 1.603 | 0.04201333 | 80.5 | 77.7 | High |
| MIF | 1.597 | 7.9902E-06 | 158.5 | 99.2 | High |
| ERLIN1 | 1.587 | 0.01843383 | 148 | 97.6 | High |
| KCNAB2 | 1.575 | 0.00057726 | 112.2 | 90.9 | High |
| IMPACT | 1.552 | 0.00888483 | 431.9 | 93.4 | High |
| MAGOHB | 1.547 | 0.02824495 | 106.8 | 71.8 | High |
| ITPA | 1.538 | 1.6608E-05 | 166.1 | 88 | High |
| UBE2I | 1.521 | 0.00011548 | 99.9 | 89.3 | High |
| FADD | 1.507 | 0.01767791 | 156.1 | 87.2 | High |
| LCN2 | 1.475 | 0.03656515 | 142.8 | 99.5 | High |
| PLIN2 | 1.466 | 0.00026487 | 174.9 | 99.7 | High |
| SPCS1 | 1.458 | 0.01759851 | 149.8 | 98.6 | High |
| GOLT1B | 1.456 | 0.02208771 | 291.2 | 200 | High |
| TOR3A | 1.455 | 0.00479504 | 225 | 99.8 | High |
| RAB1A | 1.446 | 6.3662E-05 | 127.2 | 97 | High |
| ASAP2 | 1.441 | 0.00188561 | 139.5 | 96.8 | Medium |
| NME1 | 1.44 | 0.01178699 | 135.8 | 91.5 | High |
| MRPS33 | 1.411 | 0.02797597 | 121.7 | 86.2 | High |
| NCLN | 1.375 | 0.01291237 | 121.1 | 93.2 | High |
| FTL1 | 1.367 | 0.00076046 | 126 | 99.8 | High |
| CD14 | 1.362 | 0.00123337 | 140.7 | 99 | High |
| APBB1IP | 1.36 | 0.007835 | 118.5 | 99.4 | High |
| PDXK | 1.33 | 0.00336111 | 167.8 | 96.8 | High |
| CD44 | 1.322 | 0.00536522 | 127.1 | 99.8 | High |
| KRT10 | 1.309 | 0.01368055 | 125.9 | 99.9 | High |
| RTRAF | 1.301 | 0.01533075 | 214.8 | 96.2 | High |
| RPLP2 | 1.299 | 0.0114497 | 113.1 | 96.8 | High |
| MKLN1 | 1.294 | 0.03994319 | 135.7 | 97.8 | High |
| RRBP1 | 1.29 | 0.03679254 | 512.5 | 98.6 | High |
| MTX2 | 1.289 | 0.03616002 | 136 | 100 | High |
| ANXA7 | 1.281 | 0.01450512 | 120.1 | 99.6 | High |
| RHOC | 1.27 | 0.02170953 | 122.6 | 98.8 | High |
| PAM16 | 1.264 | 0.04296765 | 106.2 | 95.8 | High |
| EIF4G2 | 0.733 | 0.04401257 | 72.9 | 99.8 | High |
| WDR77 | 0.726 | 0.04208133 | 62.6 | 97.8 | High |
| IRF5 | 0.714 | 0.04598378 | 59.9 | 94.3 | High |
| ADH7 | 0.711 | 0.04108169 | 41.9 | 91.8 | High |
| DDRGK1 | 0.709 | 0.04879336 | 70.3 | 99.2 | High |
| **BAG1** | **0.704** | **0.02914574** | **70.1** | **98.8** | **High** |
| ABCF1 | 0.701 | 0.0244954 | 71.4 | 99.4 | High |
| CORO1A | 0.697 | 0.0196878 | 85.7 | 99.8 | High |
| PLXNA1 | 0.691 | 0.04926876 | 80.5 | 99.6 | High |
| TRMT6 | 0.673 | 0.03134088 | 61 | 85.8 | High |
| FUBP3 | 0.672 | 0.03100634 | 66.3 | 100 | High |
| PLOD3 | 0.662 | 0.032238 | 47.3 | 99.8 | High |
| MFF | 0.655 | 0.02115987 | 76.4 | 98.7 | High |
| PGAM2 | 0.644 | 0.02505507 | 60.8 | 94.4 | High |
| SRBD1 | 0.623 | 0.04982566 | 57.1 | 97.4 | High |
| MOSPD2 | 0.603 | 0.04015537 | 59 | 98.3 | High |
| ANKRD52 | 0.594 | 0.00359188 | 22.4 | 72 | High |
| PGPEP1 | 0.592 | 0.01579369 | 75.6 | 99.2 | High |
| TAF6L | 0.586 | 0.00057726 | 56.1 | 95.8 | High |
| NUP35 | 0.575 | 0.00104783 | 81.2 | 98.1 | High |
| ZCCHC8 | 0.566 | 0.00575964 | 35.5 | 95.3 | High |
| UBLCP1 | 0.565 | 0.04201333 | 74.1 | 93.5 | High |
| FOXK1 | 0.559 | 0.02299553 | 49.2 | 99.8 | High |
| DPH6 | 0.559 | 0.02691072 | 64.7 | 98.8 | High |
| MAN2B1 | 0.546 | 0.00019801 | 39.9 | 99.7 | High |
| L2HGDH | 0.535 | 0.00131231 | 53.9 | 94.5 | High |
| TMX4 | 0.534 | 0.00109429 | 112 | 90.7 | High |
| NUP62 | 0.522 | 0.01023364 | 104.4 | 200 | High |
| TNRC6A | 0.515 | 0.000758 | 103 | 200 | Low |
| BMP2K | 0.515 | 0.01249039 | 48.7 | 94.6 | High |
| EPM2AIP1 | 0.505 | 0.01359808 | 44.5 | 88.1 | High |
| RNF31 | 0.505 | 0.0263966 | 101.1 | 200 | High |
| FYCO1 | 0.49 | 0.01193106 | 108.2 | 200 | High |
| CFL2 | 0.482 | 0.00315836 | 47.9 | 99.5 | High |
| SCCPDH | 0.479 | 0.00789065 | 46.2 | 96.5 | High |
| RCOR1 | 0.478 | 0.00720649 | 97.9 | 200 | High |
| POLR1B | 0.476 | 0.01291237 | 95.3 | 200 | High |
| ABI3 | 0.468 | 0.02451673 | 93.5 | 200 | High |
| RPL39 | 0.467 | 0.00052986 | 46.7 | 100 | High |
| WLS | 0.458 | 8.0023E-05 | 57.2 | 97.1 | High |
| FTO | 0.458 | 0.03482247 | 44.4 | 97 | High |
| IRF3 | 0.455 | 0.00614554 | 47 | 200 | High |
| SLC11A2 | 0.452 | 0.0042217 | 18.8 | 41.7 | High |
| UBE2F | 0.452 | 0.0097351 | 45.4 | 61.1 | High |
| ATP11C | 0.451 | 0.0239151 | 90.2 | 200 | High |
| MFN1 | 0.447 | 0.0001755 | 92.3 | 200 | High |
| ARID3A | 0.44 | 0.02942096 | 88 | 200 | Medium |
| BOP1 | 0.426 | 0.00107554 | 138.1 | 93.6 | High |
| CROT | 0.406 | 0.02942096 | 81.2 | 200 | High |
| AHSA2 | 0.401 | 1.4965E-05 | 80.1 | 200 | High |
| UNC93B1 | 0.4 | 0.0001291 | 118.8 | 79.1 | High |
| PDE4DIP | 0.399 | 0.00767187 | 21.5 | 53.8 | Low |
| CNOT11 | 0.398 | 0.00498304 | 39.3 | 98.7 | High |
| KIAA1210 | 0.398 | 0.04505673 | 79.7 | 200 | Low |
| DVL2 | 0.397 | 0.04251296 | 79.4 | 200 | Low |
| TUT4 | 0.396 | 0.00214946 | 79.2 | 200 | High |
| FNDC3A | 0.392 | 0.00012622 | 19.1 | 82.7 | High |
| STX16 | 0.391 | 0.03133431 | 39 | 99.6 | High |
| DHRS13 | 0.39 | 0.01417139 | 78 | 200 | Medium |
| STK32C | 0.389 | 0.0186762 | 77.8 | 200 | Low |
| CDR2L | 0.387 | 0.02926357 | 38.7 | 100 | Low |
| RLIM | 0.383 | 0.00616311 | 76.7 | 200 | High |
| HIGD1A | 0.37 | 0.00175832 | 31.7 | 85.6 | High |
| CCS | 0.364 | 0.00324566 | 32.3 | 99.4 | High |
| NUMA1 | 0.361 | 4.5635E-06 | 72.2 | 200 | High |
| MED6 | 0.357 | 0.01341401 | 71.5 | 200 | Low |
| CYB5B | 0.343 | 3.7474E-08 | 32.8 | 95.6 | High |
| HNRNPDL | 0.339 | 0.00113307 | 29.4 | 86.7 | High |
| N4BP1 | 0.339 | 0.00226294 | 20.3 | 59.8 | High |
| PCDHGB7 | 0.338 | 0.02286708 | 33.5 | 99 | Medium |
| POLR2K | 0.337 | 0.00090881 | 128.5 | 200 | High |
| ARF3 | 0.336 | 0.00019141 | 67.1 | 200 | High |
| CREG1 | 0.333 | 5.677E-16 | 169.2 | 200 | High |
| PCBD2 | 0.329 | 0.00385586 | 65.7 | 200 | High |
| PIGB | 0.327 | 0.00033674 | 65.3 | 200 | High |
| NDUFAF1 | 0.314 | 0.00391507 | 62.7 | 200 | High |
| RDH14 | 0.298 | 0.00026142 | 59.6 | 200 | High |
| FLRT2 | 0.294 | 8.0067E-10 | 58.7 | 200 | High |
| JPT1 HN1 | 0.286 | 0.00011977 | 57.2 | 200 | High |
| AKAP10 | 0.281 | 5.1892E-06 | 56.2 | 200 | High |
| SEPSECS | 0.281 | 0.00296611 | 27.2 | 96.8 | High |
| IRF2BP1 | 0.279 | 0.00065402 | 27 | 96.7 | High |
| ATP6V0D2 | 0.268 | 6.7051E-05 | 20.3 | 75.9 | High |
| SUPT20 | 0.262 | 9.3207E-06 | 52.5 | 200 | High |
| UXT | 0.26 | 5.677E-16 | 9 | 94.9 | High |
| GBA2 | 0.258 | 0.00017201 | 51.5 | 200 | Medium |
| UBE2Z | 0.25 | 5.677E-16 | 24.8 | 99.5 | High |
| PRKCI | 0.245 | 2.9805E-06 | 24.4 | 99.6 | High |
| SLC38A2 | 0.242 | 9.9362E-09 | 48.5 | 200 | High |
| DYM | 0.242 | 8.8521E-06 | 48.4 | 200 | High |
| CASP9 | 0.239 | 8.24E-07 | 501.4 | 200 | High |
| TAS2R106 | 0.231 | 0.00023062 | 8.5 | 45.7 | High |
| TRIM56 | 0.226 | 6.4684E-13 | 45.2 | 200 | High |
| METTL3 | 0.219 | 1.109E-07 | 19.3 | 88.2 | High |
| MAP2K2 | 0.215 | 5.677E-16 | 126.9 | 200 | High |
| FAM20B | 0.21 | 5.677E-16 | 7.5 | 48.5 | High |
| ORMDL1 | 0.207 | 1.8884E-08 | 41.3 | 200 | High |
| PANX1 | 0.207 | 1.9715E-07 | 14.5 | 70.2 | High |
| KRAS | 0.189 | 5.677E-16 | 37.8 | 200 | High |
| KDELR2 | 0.16 | 2.1261E-11 | 32 | 200 | High |
| KRT79 | 0.157 | 5.677E-16 | 31.4 | 200 | High |
| EBP | 0.157 | 5.677E-16 | 15.7 | 99.9 | High |
| TMEM132D | 0.138 | 6.3876E-13 | 27.6 | 200 | Low |
| PRCC | 0.121 | 3.5961E-11 | 24.2 | 200 | High |
| SYNE2 | 0.117 | 3.6271E-14 | 23.4 | 200 | High |
| TRIP10 | 0.104 | 5.677E-16 | 24.1 | 97.6 | High |
| CACNA1I | 0.102 | 5.062E-11 | 20.4 | 200 | Medium |
| HHIPL2 | 0.096 | 5.677E-16 | 19.1 | 200 | Medium |
| UBR1 | 0.086 | 5.677E-16 | 32.7 | 200 | High |
| OLFR607 | 0.057 | 5.677E-16 | 1.8 | 31.8 | Low |
| TRMT44 | 0.033 | 5.677E-16 | 3.3 | 99.5 | High |
